# Modular scheduling of tightly coupled production lines

**DOI:** 10.1007/s10845-025-02766-3

**Published:** 2026-05-08

**Authors:** Joan Marcè i Igual, Marc Geilen, Mitra Nasri, Twan Basten

**Affiliations:** Eindhoven University of Technology, Eindhoven, The Netherlands

**Keywords:** Scheduling, Distributed decision making, Flow shops, Modular production lines, Consensus, Constraint graphs, Collaborative manufacturing

## Abstract

Optimising productivity of tightly coupled production lines in, for instance, the production printing or semiconductor industry is difficult due to the diversity of products resulting in different product flows, the variety of constraints, and the precise timing required to coordinate multiple tightly coupled machines. A modular setup provides flexibility and cost reduction through reuse of machinery and schedulers. However, the scheduling of a modular production line is challenging as it leads to a distributed decision process where each module has a limited view of the entire system, while local scheduling decisions have a global impact on schedule feasibility. This work proposes a distributed scheduling method for tightly coupled modular sequential production lines where products cannot overtake each other during production. We develop a multi-agent framework in which a system agent propagates timing constraints of the schedulers of different modules. The system agent aims to reach consensus about the handover times of jobs between modules to converge to a globally feasible schedule. The system agent interacts with local agents that interface with local schedulers, allowing the use of existing local schedulers without modification. We illustrate the approach on production lines with multiple re-entrant flow-shop instances with setup times and due dates, which results in a challenging scheduling problem. The performance of the proposed distributed scheduling method is assessed using a monolithic exact scheduler (implemented as a constraint program, not applicable to modular problems) as a reference. It is found to deliver good quality schedules, having only a 1.15 larger makespan on average than the hypothetical optimum provided by the exact scheduler.

## Introduction

### Opportunity

High quality, productive production lines, such as the ones found in the printing or semiconductor industry, require complex processes and advanced machinery (Traganos et al., 2020; Pan et al., 2018). Due to diversity in products and different flows, the scheduling problem is very challenging (Zhang & Wang, 2016). Given the complexity of these production lines, their integration capabilities can be improved with a modular setup. Modularity allows to flexibly build the desired production line by combining the right modules, possibly of different manufacturers. Reusing modules and schedulers can also reduce costs and design effort because it does not require a specific monolithic design for a production line. Furthermore, each manufacturer can focus on optimising their modules, including their local schedulers.

Production lines may be built from loosely coupled or tightly coupled machines. In loosely coupled production lines, the product flow between machines is decoupled through buffers. In *tightly coupled* production lines, multiple machines are integrated with limited or no buffering in between them. This reduces the overall cost of the manufacturing equipment and potentially increases productivity. However, productive scheduling of tightly-coupled production lines is more difficult than scheduling loosely coupled production lines. In tightly coupled lines, the schedule of each module must account for product handover times and constraints of neighbouring modules. Local scheduling decisions have a *global impact* on schedule feasibility and productivity.

Realizing *modularity and reusability of machines* for tightly coupled production lines is challenging. Since modules must be able to operate in different production lines and production-line configurations, scheduling must follow a distributed decision-making process built upon the local schedulers of the individual modules (Zhang & Wang, 2016). Distributed scheduling is an essential enabler for flexible, customizable, multi-vendor setups, with reusable manufacturing equipment. The key challenge in distributed scheduling for tightly coupled production lines built from reusable machines is to find productive globally feasible schedules, coordinating precise handovers between machines, without having a global view of and control over the local scheduling of the individual modules. Multi-agent technology provides a potential solution to address this challenge of distribution in tightly coupled production lines.

**Fig. 1:**
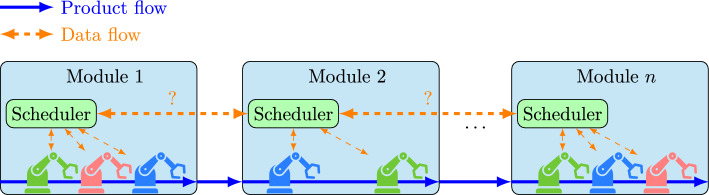
Production line composed of different modules

Figure 1 shows a tightly coupled production line consisting of multiple modules, each with its own local scheduler and consisting in turn of one or more machines. Such a production line requires a method to synchronise the modules. In this work, we propose a distributed scheduling method that allows modules to find a consensus on their local schedules that leads to a feasible global schedule.

### Contributions

Our work is the first to provide a modular scheduling approach for tightly coupled modular production lines with modules, possibly from different vendors, where each module has it own local scheduler. It applies to non-cyclic sequential module patterns processing jobs with a fixed output order. Our approach supports the integration and reuse of the modules and their local schedulers with minimal or no buffering in between modules, under minimal assumptions. Our method (explained in Sect. “Distributed scheduler framework”) provides an abstraction of a module’s output behaviour, in the form of timing bounds, the translation and propagation mechanism of constraints to other modules, as well as the necessary steps to converge to a globally feasible schedule. We implement this mechanism as a cooperative communicating multi-agent system (MAS) using a hierarchical architecture (Glavic, 2006) with local agents (LAs) for the modules and a single system agent (SA). Each LA interacts with a single module and its scheduler while the SA forwards and translates module constraints between local agents. Our design aims to minimise the assumptions on the behaviour of local schedulers, so that it can be flexibly integrated with existing local schedulers. By following an iterative approach and a translation of constraints, our method guarantees that if a consensus between modules is found, then the local schedules together form a global schedule that is feasible.

The approach is illustrated on a production line with multiple 2-re-entrant machines, where products are processed two times in the same machine. Re-entrant production lines can be found in semiconductor lithography or production printing, for instance (Zhang & Wang, 2016). We model the machines as re-entrant flow shops with setup times, due dates, no overtaking between machines, and fixed output order of jobs. The setup times and due dates allow modelling of travelling times and limited buffering by expressing lower and upper bounds between operations, respectively. This makes the approach suitable for tightly coupled modules. The scheduling goal is makespan minimization. The essential (local) scheduling decisions are made by choosing the order of operations of each re-entrant machine. We integrate our MAS with two different existing local schedulers for 2-re-entrant machines (van Pinxten et al., 2017; Jeong & Kim, 2014) for experimental analysis, showing feasibility of reusing existing schedulers, also in combination within a single production line.

We evaluate the quality of our distributed scheduling solution using as a reference a monolithic (all-knowing) scheduler implemented as a constraint program in a constraint solver. We observe a good trade-off between schedule quality and scheduling speed by generating schedules for large systems with 10 modules and 300 jobs at an average of 1.5 s per job with a makespan only 1.15 times larger than the makespan produced by the monolithic scheduler (that cannot be used in modular setups).

The paper is structured as follows, Sect. “Related work” gives an overview of the related work. Section “Flow shops with output order constraints” defines the flow-shop problem and our system model. Section “Production-line problem” explains how modules are composed into a production line. Section “Distributed scheduler framework” presents our multi-agent solution to the distributed scheduling problem. Section “Design decisions” explains the reasoning behind the design choices. Section “Experimental evaluation” has the experimental evaluation. Section “Conclusions” concludes.

## Related work

Our work focuses on tightly coupled production lines, as a combination of multiple re-entrant flow-shops and their schedulers, with no or limited (work-in-progress) buffering in-between modules. We start discussing approaches to distributed manufacturing and satisfying handover constraints. Then, we give details on existing approaches for re-entrant flow-shop scheduling with added constraints, including work-in-progress buffering constraints. Finally, we look at MAS scheduling as a way to handle different schedulers (or agents) in a production line. Thus, motivating our decision to use a coordinating MAS for this problem.

### Distributed systems

Distributed manufacturing involves producing different parts of a product in different factories or on different machines, usually distributed geographically or physically. An important advantage of distribution is to create multiple possible product flows, allowing more customisation and personalisation (Sadiku et al., 2023). Typically, manufacturing of products has been modelled with variants of the job-shop or flow-shop scheduling problems (JSSP, FSSP) (Emmons & Vairaktarakis, 2013). For distributed manufacturing, there are two categories of problems: the distributed job-shop/flow-shop scheduling problem (DJSP, DFSP) (Bagheri Rad & Behnamian, 2022) and the job-shop scheduling problem with transportation resources (JSPT) (Nouri et al., 2016).

In the basic form of the DJSP and DFSP, multiple factories are available to process a job. But the entire job must be processed in a single factory. Thus, the scheduling problem consists of choosing the factory where a job will be made and the ordering of the jobs inside the factory (Naderi & Ruiz, 2010; Jia et al., 2002). Although the basic DJSP/DFSP consider distribution across factories, the techniques also apply to manufacturing systems where jobs are distributed over multiple machines in a single factory, as long as jobs should be allocated to a single machine in such a factory. These techniques are therefore not applicable to modular setups where jobs need to be scheduled on multiple machines.

Work on transportation between factories in DJSP/DFSP is rare given the assumption that jobs are processed entirely in a single factory (Lohmer & Lasch, 2021). Chan et al. (2012) considered transportation using a centralised agent that uses Tabu search and simulated annealing to generate a schedule. More recently, Ziaee (2017) proposed a centralised heuristic approach to solve the DJSP with transport times as well as transfer of work in progress (WIP) between factories. These approaches address *loosely* coupled production systems. Moreover, given that the approaches are *centralized*, they do not apply to modular setups as they assume full information about and control over the entire production process.

The JSPT defines a job-shop problem with the transportation resources required to transport elements (robots, vehicles...) from one machine to another and their availability. There are both heuristics and exact approaches for JSPT (Nouri et al., 2016). However, these approaches cannot be applied to modular production lines due to their central nature that assumes full information and control.

The systems considered in this work, tightly coupled modular production lines, are another form of distributed manufacturing, focused on a single manufacturing facility. These production lines have multiple modules, each with their own local schedules of operations to be performed, requiring coordination of work-in-progress handover times. A common assumption in DFSP and DJSP is the availability of unlimited (work-in-progress) buffering between modules (Chan et al., 2012; Ziaee, 2017). However, in tightly coupled production lines with only limited buffering between modules, the handover times between modules are critical. Handovers between machines are bounded in duration from below by transportation time and from above by limited buffering. In *tightly coupled* setups, the modules need to achieve *consensus* about the handovers. For *modular* setups, this consensus needs to be achieved in a *distributed* manner, because it cannot be assumed that the production-line scheduler has control over scheduling decisions of individual machines.

### Flow-shop problems

Inspired by the FSSPs in wafer fabrication, TFT-LCD manufacturing, or production-printing problems (Rifai et al., 2015; Waqas et al., 2015), in this paper, we focus on production lines with fixed job completion order (Lin & Hwang, 2011), precedence constraints between operations (Balas et al., 2008), and without overtaking in-between machines (Gupta & Stafford, 2006). We illustrate our framework in a production line with re-entrant flow shops (Riezebos et al., 1995), where a job is processed multiple times by a machine. Because overtaking is not allowed and the output order is fixed, without re-entrance, the scheduling problem is trivial as there are no decisions to make.

Online schedulers take decisions in little time to allow for a continuous production. There exists an online scheduler with two different variations for 2-re-entrant flow-shop problems: the bounded heuristic constraint-based scheduler (BHCS) and the multi-dimensional bounded heuristic constraint-based scheduler (MD-BHCS) (van Pinxten et al., 2017). They are based on the heuristic constraint-based scheduler (HCS) (Waqas et al., 2015), that uses a constraint-graph model to check for feasibility of candidate schedules and to determine earliest start times of operations. These heuristics can also solve variable re-entrant problems, where jobs can have a different number of re-entrances in each machine (van der Tempel et al., 2018).

Offline scheduling solutions for the same problem are the modified FL (MFL) or modified NEH (MNEH) heuristics (Jeong & Kim, 2014), which use a branch-and-bound algorithm to schedule 2-re-entrant flow-shops. They cannot be used for online scheduling, as the execution time increases significantly with the number of jobs.

All these solutions are designed to work in a monolithic production line with a single re-entrant machine and a single scheduler. This work aims to schedule a production line composed of multiple re-entrant flow shops by coordinating existing schedulers for re-entrant flow shops. Thus, our work allows the scheduling of multiple re-entrant machines in a system of systems.

We assume that our production lines are tightly coupled. This means that there is limited or no buffering (also known as limited WIP) between the modules of the production line. There are many existing solutions that consider this type of constraints. The previously mentioned BHCS andMD-BHCS can handle them due to their constraint-graph-based modelling and analysis. Liu et al. (2018) designed two monolithic heuristics that can also handle the limited or no buffering scenarios. Zhang et al. (2017) present a heuristic for a two-stage production line combining batch processing and processing of individual jobs, with limited buffering between the two stages. Qin et al. (2021) designed a genetic algorithm for hybrid flow shops with limited waiting time. However, all these solutions focus on monolithic central approaches. Our work is the first to consider modular scheduling of tightly coupled systems.

### Multi-agent approaches

A MAS is a collection of computerised agents. Each agent can implement different behaviours to solve coordination problems. For manufacturing, MAS can model different machines or modules and abstract part of their behaviour in a common framework. Seitz et al. (2021) propose such a framework, where different manufacturing facilities are modelled as agents and place a bid to decide who produces a product. Their approach is suitable for reusing local schedulers, but not for tightly-coupled production lines, as the agents communicate with each other to allocate production of products based on the local module status but they do not consider handover constraints.

Different MAS architectures are available for scheduling (Glavic, 2006). Common approaches use one agent per operation or per constraint and resolve conflicts either through a bidding process or with a central agent until none are remaining (Fazlirad & Brennan, 2018; Maturana & Norrie, 1996). This approach is also applied to solve distributed constraint optimisation problems (Fioretto et al., 2018; Liu & Sycara, 1993), like our problem. However, since we want to reuse existing schedulers, we cannot use approaches that actively decide the start time of the operations in different modules and we look at heterarchical MAS architectures (Toptal & Sabuncuoglu, 2010). Our architecture is a cooperative MAS with multiple LAs interacting with the local schedulers and a single SA propagating and translating their messages. Since this SA is not scheduling, we consider it a heterarchical architecture from the scheduling point of view and a hierarchical architecture from the organisation point of view.

Heterarchical MAS scheduling approaches usually use iterative refinement (Toptal & Sabuncuoglu, 2010). Kouider and Bouzouia (2011) propose a hierarchical architecture (heterarchical from the scheduling point of view) where the SA decomposes the problem into smaller problems, one per machine, and each machine has a LA. The LAs exchange completion times of operations and as soon as the dependencies of an operation are satisfied, the LA schedules it. In contrast, our heterarchical architecture also employs iterative refinement but assumes that the decomposition is derived from the linking of multiple modules, each possibly composed of multiple machines and each with its own local scheduler. Another iterative approach is proposed by Liu and Sycara (1993). They create a heterarchical MAS for constraint satisfaction in job-shop scheduling with due dates. Their architecture has order agents, managing jobs, and resource agents, managing machines. Order agents only talk to resource agents and vice versa until there are no more conflicts. The information exchanged between agents is, primarily, intervals on the start times of the operations. This approach is similar to ours as we share bounds on the relative start times of consecutive input and output operations. A work that explicitly considers consensus in the handover times between machines is the one from Miyamoto et al. (2020). They propose a consensus mechanism for the JSSP where they minimise the discrepancy in the start times of the handover operations, reaching consensus when the error reduces to 0 on all handovers. Their solution is a heterarchical architecture where the local agents have full control of the start times of the operations.

Our approach differs from Kouider and Bouzouia (2011), Liu and Sycara (1993) and Miyamoto et al. (2020) by enabling modularity and reuse of local schedulers. The three discussed methods rely on control of the local scheduler. In contrast, our approach focuses on reaching consensus on handovers only, leaving the scheduling of operations within modules to local schedulers. The mentioned approaches do not support modularity, as their agents rely on specifying the start times of all the operations.

As an alternative for heterarchical MAS scheduling approaches, Zhang and Wang (2016) propose a *hierarchical* MAS architecture with different agents for different types of machines and jobs. Their approach divides the architecture in a system layer and machine layer. The system layer models the capacity of each machine and generates a plan for the number of products processed in a period of time. The machine layer receives the number of products that must be produced in a period and generates a schedule with the exact start times required for the selected period. The design handles dynamic events in the machine layer but it is not suitable for tightly coupled production lines where the timing of one module greatly impacts the scheduling possibilities of other modules.

In summary, our method for scheduling jobs on production lines is the first that supports modularity in the presence of tight coupling between modules. This allows integration of modules, possibly from different original equipment manufacturers (OEMs), while reusing their local schedulers.

## Flow shops with output order constraints

In this section, we define the flow-shop scheduling problem that we assume as an appropriate abstraction of the scheduling problem of every module and give the definition of a feasible schedule. We further explain the constraint-graph model used to check for feasibility and we introduce an illustrative example.

### Flow-shop model

A flow shop with setup times, relative due dates, and a fixed output order is a tuple

$$\begin{aligned} \tau = (J, M, \mathsf{ops}, \mathsf{mch}, \mathsf{prc}, \mathsf{si}, \mathsf{sd}, \mathsf{dd}) \end{aligned}$$

$J = \{j_{1},\ldots ,j_{|J|}\}$ is the set of jobs to be processed. For every job $j_{u}$, the index $u$ in $\mathbb {N}$ with $1 \le u \le |J|$ denotes its place in the output order. $M$ is the set of machines on which the jobs are processed. $\mathsf{ops}(j_{u}) = \{o_{u,1},\ldots ,o_{u,|\mathsf{ops}(j_{u})|}\}$ is the set of operations performed by a job $j_{u}$ in $J$; these sets are disjoint for different jobs. For every operation $o_{u,k}$, the index $k$ in $\mathbb {N}$ with $1 \le k \le |\mathsf{ops}(j_{u})|$ determines its place in the execution order of the operations of job $j_{u}$.

The set $O$ of all operations is defined as $O = \bigcup _{j\in J}\mathsf{ops}(j)$. The function $\mathsf{mch} : O \rightarrow M$ defines which machine performs each operation. We define the auxiliary functions $\mathsf{fst}(j_{u}) = o_{u,1}$ and $\mathsf{lst}(j_{u}) = o_{u,|\mathsf{ops}(j_{u})|}$ to refer to the first and last operations of a job $j_{u}$, respectively. These functions help defining restrictions on the flow shop. Depending on the restrictions, different flavours of flow shop can be obtained. If a job can visit a machine multiple times, it is called a re-entrant flow shop and if it can only visit one machine at most two times, it is a 2-re-entrant flow shop.    

The processing time of an operation on a machine is defined as $\mathsf{prc} : O \rightarrow \mathbb {R}_{> 0}$. We define setup times as the delay between the completion of an operation and the start of another one. In practice, setup times often model product travel times or machine or product preparation times. We distinguish two types of setup times: sequence-independent and sequence-dependent setup times. Sequence-independent setup times $\mathsf{si} : O \times O \hookrightarrow \mathbb {R}_{\ge 0}$ are always-enforced constraints such as travel times between machines. If $\mathsf{si}(o_{1}, o_{2})$ is undefined, no restriction exists between the completion of $o_{1}$ and the start of $o_{2}$. Sequence-dependent setup times $\mathsf{sd} : O \times O \hookrightarrow \mathbb {R}_{\ge 0}$ model preparation times, such as the time needed for an arm to reach a given position. If the arm is already at the desired position, then the preparation time is shorter. Thus, $\mathsf{sd}$ depends on the sequence of operations that a machine performs. The $\mathsf{sd}(o_{1}, o_{2})$ constraint only applies if $o_{1}$ is scheduled immediately before $o_{2}$ on the same machine. We assume that it is defined between all operations mapped onto the same machine. The last element of the tuple is the relative deadline, or due date, $\mathsf{dd} : O \times O \hookrightarrow \mathbb {R}_{\ge 0}$, that defines the maximum time delay between the completion time of one operation and the start time of another one. If $\mathsf{dd}(o_{1}, o_{2})$ is undefined, there is no due date between $o_{1}$ and $o_{2}$.

### Feasible schedules

A schedule $\Omega : O \rightarrow \mathbb {R}_{\ge 0}$ describes the start times for all the operations in $O$ and is a potential solution of the flow-shop problem. It is called **feasible** iff the following constraints are met:

1. Every job must perform its operations in the order specified by the operations’ indices:
  $$\begin{aligned} \begin{aligned}&\forall u, k \in \mathbb {N} \wedge 1 \le u \le | J | \wedge 1 \le k < |\mathsf{ops}(j_{u})|:\\&\qquad \Omega (o_{u,k}) + \mathsf{prc}(o_{u,k}) \le \Omega (o_{u,k+1}) \end{aligned} \end{aligned}$$
2. Every machine can only perform one operation at a time:
  $$\begin{aligned} \begin{aligned}&\forall o_{1}, o_{2} \in O: \mathsf{mch}( o_{1}) = \mathsf{mch}( o_{2}) \wedge o_{1}\ne o_{2}\\&\quad \Rightarrow \begin{aligned}&\Omega (o_{1}) + \mathsf{prc}(o_{1}) \le \Omega (o_{2}) \vee \Omega (o_{2}) + \mathsf{prc}(o_{2}) \le \Omega (o_{1}) \end{aligned} \end{aligned} \end{aligned}$$
3. If two operations are scheduled on the same machine and no other operation starts between the start of $o_{1}$ and the start of $o_{2}$, then the sequence-dependent setup times must be met:
  $$\begin{aligned} \begin{aligned} \forall o_{1}, o_{2}\in O: &\, \begin{aligned}[t] \Big (&\mathsf{mch}( o_{1}) = \mathsf{mch}( o_{2}) \wedge \Omega (o_{1})< \Omega (o_{2}) \wedge {}\\&\big ({\not \exists} o \in O: \mathsf{mch}(o) = \mathsf{mch}( o_{1}) \wedge \Omega (o_{1})< \Omega (o) < \Omega (o_{2}) \big )\Big ) \end{aligned}\\&\Rightarrow \Omega (o_{1}) + \mathsf{prc}(o_{1}) + \mathsf{sd}(o_{1}, o_{2}) \le \Omega (o_{2}) \end{aligned} \end{aligned}$$
4. If the sequence-independent setup time between two operations is defined, it must be respected^1^:
  $$\begin{aligned} \forall ( o_{1}, o_{2}) \in {{\,\textrm{dom}\,}}(\mathsf{si}):\Omega (o_{1}) + \mathsf{prc}(o_{1}) + \mathsf{si}(o_{1}, o_{2}) \le \Omega (o_{2}) \end{aligned}$$
5. If a due date is defined between two operations $o_{1}$ and $o_{2}$, then $o_{2}$ cannot start later than allowed by the relative due date:
  $$\begin{aligned} \forall ( o_{1}, o_{2}) \in {{\,\textrm{dom}\,}}(\mathsf{dd}):\Omega (o_{1}) + \mathsf{prc}(o_{1}) + \mathsf{dd}(o_{1}, o_{2}) \ge \Omega (o_{2}) \end{aligned}$$
6. Jobs must complete in the order specified by their indices:
  $$\begin{aligned} \forall u \in \mathbb {N} \wedge 1 \le u < | J |:\ \Omega (\mathsf{lst}(j_{u})) \le \Omega (\mathsf{lst}(j_{u+1})) \end{aligned}$$
7. A job cannot overtake other jobs in-between machines:
  $$\begin{aligned} \begin{aligned}&\forall u, v, k, l \in \mathbb {N} \wedge 1 \le u, v \le | J | \wedge u \ne v \wedge{} \\& \quad 1 \le k< |\mathsf{ops}(j_{u})| \wedge 1 \le l< |\mathsf{ops}(j_{v})|:\\&\quad \begin{aligned}& \begin{aligned}&\big((\mathsf{mch}( o_{u, k}) = \mathsf{mch}( o_{v, l}) \wedge \mathsf{mch}( o_{u, k+1}) = \mathsf{mch}( o_{v, l+1})\\&\wedge \Omega (o_{u,k}) < \Omega (o_{v,l}) \big ) \Rightarrow \Omega (o_{u,k+1}) \le \Omega (o_{v,l+1}) \end{aligned} \end{aligned} \end{aligned} \end{aligned}$$

### Constraint-graph model and feasibility check

We use, without loss of generality, the constraint-graph model (Waqas et al., 2015) to represent the constraints of a production line for a given order of operations on each of the machines, to test the feasibility of a schedule and to obtain the start times of its operations. We define sequences of operations on the machines $\mathsf{seq} : M \rightarrow O^+$, where $\mathsf{seq}(m)(i)$ denotes the $i$-th operation that a machine $m$ must perform. We assume that machine sequences $\textsf{seq}$ specify sequences for all machines in the flow shop, ordering all operations allocated to each of these machines. A constraint-graph model $\mathcal {G}_{\mathsf{seq}} = (V, E)$ is a labelled directed graph built from the problem $\tau$ and the machine sequences $\textsf{seq}$.    

In the constraint graph $$\mathcal {G}_{\mathsf{seq}}$$, all operations are represented as vertices ($$V = O $$). An edge $$( o_{1}, b, o_{2})\in E$$ labelled with a value $$b$$ represents the constraint $$\Omega (o_{1}) + b \le \Omega (o_{2})$$ between $$o_{1}$$ and $$o_{2}$$. The graph is built by representing every constraint defined in Sect. “Feasible schedules” with a set of labelled edges. After the machine sequences are given, all constraints are fixed. In particular, the sequence-dependent setup-time constraints can be determined. Constraints are mapped as follows:

- Constraint C1: job operation order. For every pair $o_{u, k}$ and $o_{u, k+1}$ of operations in $\mathsf{ops}(j_{u})$ of a job $j_{u}$ in $J$, we add an edge with the value $\mathsf{prc}(o_{u,k})$ from $o_{u,k}$ to $o_{u,k+1}$.
- Constraint C2 and C3: one operation per machine at a time and sequence-dependent setup times. For each machine $m$ in $M$ and pair of consecutively scheduled operations $o_{1} = \mathsf{seq}(m)(i)$ and $o_{2} = \mathsf{seq}(m)(i + 1)$, we add an edge with the value $\mathsf{prc}(o_{1}) + \mathsf{sd}(o_{1}, o_{2})$ from $o_{1}$ to $o_{2}$. This edge represents both constraints C2 and C3.
- Constraint C4: sequence-independent setup times. For every pair $( o_{1}, o_{2})$ of operations in the domain of $\mathsf{si}$, we add an edge with the value $\mathsf{prc}(o_{1}) + \mathsf{si}(o_{1}, o_{2})$ from $o_{1}$ to $o_{2}$.
- Constraint C5: due date between operations. For every pair $$( o_{1}, o_{2})$$ of operations in the domain of $$\mathsf{dd}$$, we add an edge with the value $$-\mathsf{dd}(o_{1}, o_{2})-\mathsf{prc}(o_{1})$$ from $$o_{2}$$ to $$o_{1}$$. The edge is negative and goes in the opposite direction because C5 is in the form $$\Omega (o_{1}) + \mathsf{prc}(o_{1}) + \mathsf{dd}(o_{1}, o_{2}) \ge \Omega (o_{2})$$ while the graph only represents constraints of the form $$\Omega (o_{x}) + b \le \Omega (o_{y})$$. Thus, here $$ o_{x} = o_{2}$$, $$ o_{y} = o_{1}$$ and $$b = -\mathsf{prc}(o_{1}) -\mathsf{dd}(o_{1}, o_{2})$$.
- Constraint C6: job output order. For every pair of consecutive jobs $j_{u}$ and $j_{u+1}$ in $J$, we add an edge from $\mathsf{lst}(j_{u})$ to $\mathsf{lst}(j_{u+1})$ with value 0.
- Constraint C7: no overtaking in-between machines. For each machine $m$ in $M$ and pair of consecutive operations $o_{u, k} = \mathsf{seq}(m)(i)$ and $o_{v, l} = \mathsf{seq}(m)(i + 1)$, if they are not the last operations and their next operations are performed on the same machine, i.e., $\mathsf{mch}( o_{u, k+1}) = \mathsf{mch}( o_{v, l+1})$, we add an edge from $o_{u, k+1}$ to $o_{v, l+1}$ with the value 0.

When multiple constraints exist between two operations, these can be captured with a single edge; the label of the edge is determined by the largest value among those constraints.

Since the labelled edges of the graph represent timing constraints between operations, relative timing constraints between operations are captured by the length of the longest path that goes from one operation to the other. Thus, the feasible schedule $\Omega$ with the earliest start time of all operations is found by taking the lengths of the longest path between the first operation in the system and each of the other operations. Moreover, these longest paths, and hence a feasible schedule, only exist iff no positive cycles exist in the graph. Any such a positive cycle represents a chain of constraints that cannot be satisfied (van Pinxten et al., 2017).

#### Example 3.1

**Fig. 2:**
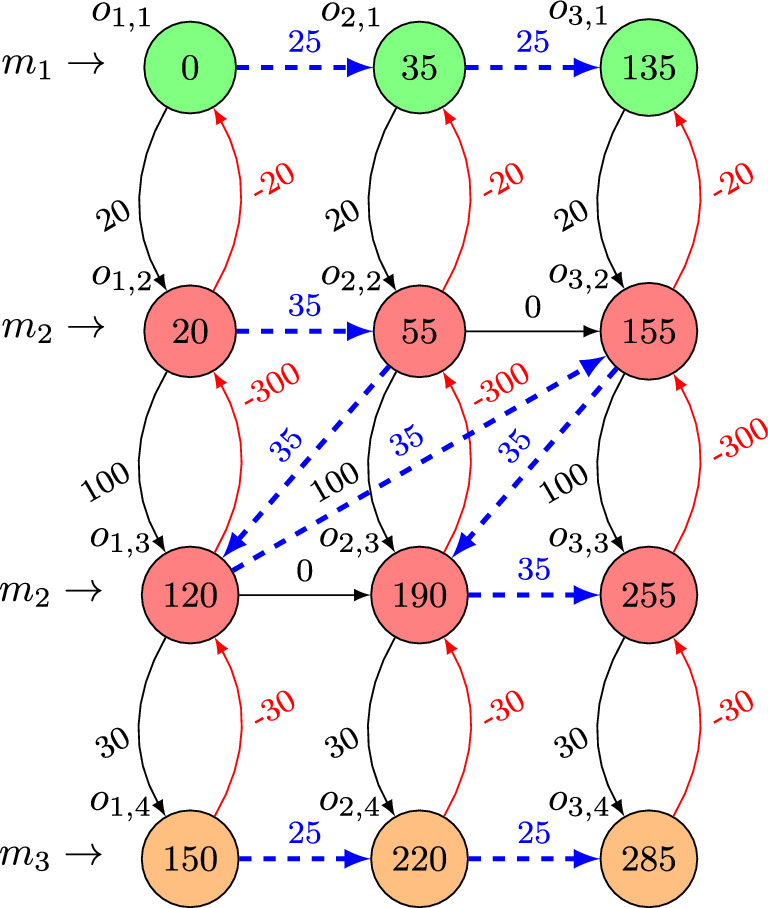
Representation of the sequences of Example 3.1 (blue dashed lines) in a constraint graph. The values inside the nodes are the start times of the earliest feasible schedule $\Omega$

An example of a re-entrant flow-shop problem is a production printer. It has three machines, $$M=\{m_{1},m_{2},m_{3}\}$$. $$m_{1}$$ is the printer input machine taking in sheets of paper, $$m_{2}$$ is the image transfer machine, that transfers ink onto the paper, and $$m_{3}$$ is the printer output machine, releasing the printed sheets. We assume that there are three sheets to be printed, represented by jobs $$ J = \{j_{1},j_{2},j_{3}\}$$. Every job has four operations ($$\mathsf{ops}(j_{u}) = \{o_{u, 1}, \dotsc , o_{u, 4}\}$$). The first operation is the input operation, then there is one image transfer operation for each side of the paper and finally there is the output operation. The mapping $$\mathsf{mch}$$ of operations as well as the $$\mathsf{prc}$$, $$\mathsf{si}$$ and $$\textsf{dd}$$ values can be seen in Table 1. If both $$o_{1}$$ and $$o_{2}$$ are performed on the same machine ($$\mathsf{mch}( o_{1}) = \mathsf{mch}( o_{2})$$), then $$\mathsf{sd}(o_{1}, o_{2}) = 5$$. Figure 2 shows a constraint-graph representation for the given flow shop for the following operation sequences for the three machines: $$\mathsf{seq}(m_{1}) $$$${}= \langle o_{{1,1}} , $$$$ o_{{2,1}} ,o_{{3,1}} \rangle $$, $$\mathsf{seq}(m_{2})$$$${}= \langle o_{{1,2}} , $$$$o_{{2,2}} ,o_{{1,3}},$$$$o_{{3,2}} , $$$$ o_{{2,3}} ,o_{{3,3}} \rangle $$, and $$\mathsf{seq}(m_{3}) $$$${}= \langle o_{{1,4}} , $$$${}o_{{2,4}} ,o_{{3,4}} \rangle $$. As the operations $$o_{u,2}$$ and $$o_{u,3}$$ of every job $$j_{u} \in J $$ are performed on the same machine, we call these *re-entrant operations*. With the constraint graph, the Bellman-Ford longest path algorithm can be used to obtain the schedule with earliest starting times by determining the lengths of the longest paths between $$o_{1,1}$$ and each of the other operations. The length of this path is the value in the center of each node in Fig. 2. The longest path from $$o_{1,1}$$ to $$o_{2,1}$$, for instance, has length $$35$$ and goes via $$o_{1,2}$$ and $$o_{2,2}$$. The schedule with the earliest start times is also the schedule with the lowest makespan for the given sequences. $$\blacksquare $$        

**Table 1:** Values for Example 3.1

(a) Values for $\mathsf{prc}$ and $\textsf{mch}$			
$o_{u,j}$	$\mathsf{prc}(o_{u,j})$	$\mathsf{mch}(o_{u,j})$	
$o_{u,1}$	20	$m_{1}$	
$o_{u,2}$	30	$m_{2}$	
$o_{u,3}$	30	$m_{2}$	
$o_{u,4}$	20	$m_{3}$	

(b) Values for $\mathsf{si}$ and $\mathsf{dd}$			
Between		$\mathsf{si}$	$\mathsf{dd}$
$o_{u,1}$	$o_{u,2}$	0	0
$o_{u,2}$	$o_{u,3}$	70	270
$o_{u,3}$	$o_{u,4}$	0	0

## Production-line problem

In this section, we explain how we compose multiple flow shops defined in Sect. “Flow shops with output order constraints” into a production line, like the one shown in Fig. 1, as a *production-line problem (PLP)*. Then, we define the feasibility conditions for its schedule. In the following sections, we explain how our MAS generates a schedule for a production line.

The way we define the composition allows also to model the whole production line as a single instance of the flow-shop problem defined in Sect. “Flow-shop model”. This is useful later in Sect. “Experimental evaluation” to compare against a monolithic scheduler. We consider non-cyclic sequential module patterns with a fixed output order of jobs. These patterns can be found for instance in production printing or the semiconductor industry.

### Composition

A production line $$P=(N, T)$$ is composed of a set $$N$$ of modules and a set $$T$$ of transfer points between modules. These transfer points model the transfer of products from one module to the next one. Every module $$n_{i} \in N$$ comes with an instance of the flow-shop problem $$\tau _{i} = (J_{i},M_{i},\mathsf{ops}_{i},\mathsf{mch}_{i},\mathsf{prc}_{i},\mathsf{si}_{i},\mathsf{sd}_{i},\mathsf{dd}_{i})$$ defined in Sect. “Flow-shop model”. The index $$i \in \mathbb {N}, 1 \le i \le |N|$$ denotes the order of the modules. Because the jobs flow from one module to the next and all jobs are processed by all modules, we assume that the set of jobs is the same for all modules, that is, $$J_{i} = J $$ for all $$i$$, and that the sets of machines $$M_{i}$$ are disjoint. Every transfer point $$T_{i} = (\mathsf{ts}_{i}, \mathsf{td}_{i})$$ in $$T$$, with $$1 \le i < |N|$$, represents a connection between modules where jobs flow from module $$n_{i}$$ to module $$n_{i+1}$$. The setup-time function $$\mathsf{ts}_{i} : J \rightarrow \mathbb {R}_{\ge 0}$$ defines the minimum transfer time of a job between leaving module $$n_{i}$$ and arriving at module $$n_{i+1}$$. Likewise, the due date $$\mathsf{td}_{i} : J \hookrightarrow \mathbb {R}_{\ge 0}$$ defines the maximum allowed transfer time of a job. If no due date is defined, then there is no maximum allowed transfer time for a job (e.g., in case of unlimited buffering where a job can stay in a buffer as much as needed).            

### Feasible schedules

A schedule $\Omega$ for a production line is a function that maps all operations of all modules to their start times, i.e., $\Omega : O \rightarrow \mathbb {R}_{\ge 0}$, with $O =\bigcup _{n_{i} \in N} O_{i}$. It can be composed from the local schedules of the modules $\Omega _{i} : O_{i} \rightarrow \mathbb {R}_{\ge 0}$. It is called feasible if all the local schedules $\Omega _{i}$ are feasible and the following constraints are respected for the transfer points:

1. Minimum transport times must be respected:
  $$\begin{aligned} \forall T_{i}& \in T, j \in J: \\ & \Omega _{i}(\mathsf{lst}_{i}(j)) + \mathsf{prc}_{i}(\mathsf{lst}_{i}(j)) + \mathsf{ts}_{i}(j) \le \Omega _{i+1}(\mathsf{fst}_{i+1}(j)) \end{aligned}$$
2. Due dates must be respected, if defined:
  $$\begin{aligned} \forall T_{i}& \in T, j \in {{\,\textrm{dom}\,}}(\mathsf{td}_{i}): \\ &\Omega _{i}(\mathsf{lst}_{i}(j)) + \mathsf{prc}_{i}(\mathsf{lst}_{i}(j)) + \mathsf{td}_{i}(j) \ge \Omega _{i+1}(\mathsf{fst}_{i+1}(j)) \end{aligned}$$

### Global constraint-graph model

The constraints of a full production-line problem and the chosen sequences $\mathsf{seq}$ of all modules can be represented as a global constraint graph $\mathcal {G}_{\mathsf{seq}}^{global} = (V, E)$, similar to the way flow-shop problems are represented in Sect. “Constraint-graph model and feasibility check”. All operations of all modules are represented as vertices ($V = O = \bigcup _{n_{i} \in N} O_{i}$). The edges between operations are defined in the same way as in Sect. “Constraint-graph model and feasibility check”. Additionally, we add edges to represent constraints CT1 and CT2:

- Constraint CT1: transfer setup times. For every $T_{i}$ in $T$ and job $j$ in $J$, we add an edge from $\mathsf{lst}_{i}(j)$ to $\mathsf{fst}_{i+1}(j)$ with the value $\mathsf{prc}_{i}(\mathsf{lst}_{i}(j)) + \mathsf{ts}_{i}(j)$.    
- Constraint CT2: transfer due dates. For every $T_{i}$ in $T$ and job $j$ in the domain of $\mathsf{td}_{i}$, we add an edge from $\mathsf{fst}_{i+1}(j)$ to $\mathsf{lst}_{i}(j)$ with the value $-\mathsf{prc}_{i}(\mathsf{lst}_{i}(j)) - \mathsf{td}_{i}(j)$.    

    

#### Example 4.1

**Fig. 3:**
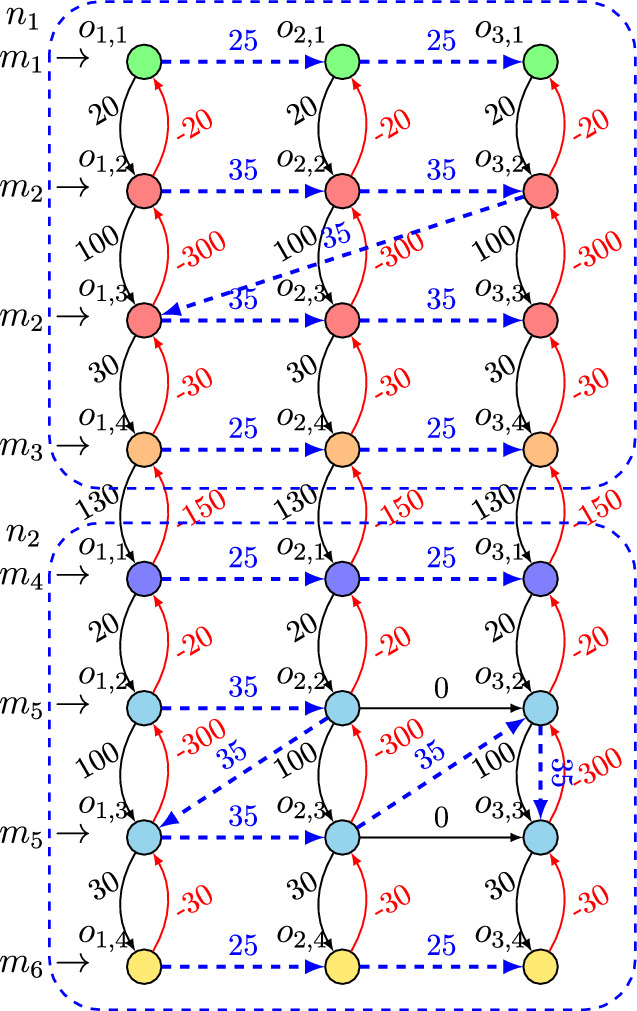
Constraint graph of Example 4.1. Columns and rows denote jobs and operations. Machines are indicated on the left, modules are represented by  rectangles, and sequences $\textsf{seq}_{i}$ by  arrows

We define a production line with two modules $$n_{1}$$ and $$n_{2}$$ each with three machines, $$M_{1} = \{m_{1}, m_{2}, m_{3}\}$$ and $$M_{2} = \{m_{4}, m_{5}, m_{6}\}$$. Both modules are instances of the flow shop of Example 3.1. We schedule three jobs $$ J = \{j_{1}, j_{2}, j_{3}\}$$, with four operations per job per module, $$\mathsf{ops}_{1}(j_{u}) = \{o_{u,1},\dotsc , o_{u,4}\}$$, $$\mathsf{ops}_{2}(j_{u}) = \{o_{u, 1}, \dotsc , o_{u, 4}\}$$ (for $$u\in \{1,2,3\}$$). The mapping $$\mathsf{mch}$$ of operations as well as the values for processing times $$\mathsf{prc}_{i}$$, sequence-independent setup times $$\mathsf{si}_{i}$$, and due dates $$\mathsf{dd}_{i}$$ (for $$i\in \{1,2\}$$) can be found in Table 2. If both $$o_{1}$$ and $$o_{2}$$ are performed on the same machine, then $$\mathsf{sd}_{i}(o_{1}, o_{2}) = 5$$ (for $$i\in \{1,2\}$$). All these values align with the values in Example 3.1. There is a transfer point $$T_{1} = (\mathsf{ts}_{1}, \mathsf{td}_{1})$$. For every job $$j_{u}$$, the transfer setup time is $$\mathsf{ts}_{1}(j_{u}) = 110$$ and the transfer due date is $$\mathsf{td}_{1}(j_{u}) = 130$$. Figure 3 shows a constraint graph for the production line with the given sequences $$\mathsf{seq}_{1}(m_{1}) $$$${}= \langle o_{1,1}, o_{2,1}, o_{3,1}\rangle $$, $$\textsf{seq}_{1}(m_{2}) $$$${}= \langle o_{{1,2}} ,o_{{2,2}} ,o_{{3,2}},$$$$o_{{1,3}} ,o_{{2,3}} ,o_{{3,3}} \rangle $$, $$\mathsf{seq}_{1}(m_{3}) $$$${}= \langle o_{{1,4}},$$$$o_{{2,4}}, $$$$o_{{3,4}} \rangle $$, $$\mathsf{seq}_{2}(m_{4}) $$$${}= \langle o_{{1,1}} , $$$$ o_{{2,1}} ,o_{{3,1}} \rangle $$, $$\mathsf{seq}_{2}(m_{5}) $$$${}= \langle o_{{1,2}} , $$$$o_{{2,2}} ,o_{{1,3}}, $$$$o_{{2,3}} , $$$$ o_{{3,2}} ,o_{{3,3}} \rangle $$, and $$\mathsf{seq}_{2}(m_{6}) $$$${}= \langle o_{{1,4}},$$$$o_{{2,4}} ,o_{{3,4}} \rangle $$ indicated by the arrows. $$\blacksquare $$                    

**Table 2:** Values for Example 4.1

(a) Values for $\mathsf{prc}_{i}$, $\mathsf{mch}_{1}$ and $\textsf{mch}_{2}$			
$o_{u,k}$	$\textsf{prc}_{i}$	$\mathsf{mch}_{1}$	$\mathsf{mch}_{2}$
$o_{u,1}$	20	$m_{1}$	$m_{4}$
$o_{u,2}$	30	$m_{2}$	$m_{5}$
$o_{u,3}$	30	$m_{2}$	$m_{5}$
$o_{u,4}$	20	$m_{3}$	$m_{6}$

(b) Values for $\mathsf{si}_{i}$ and $\mathsf{dd}_{i}$			
Between		$\mathsf{si}_{i}$	$\mathsf{dd}_{i}$
$o_{u,1}$	$o_{u,2}$	0	0
$o_{u,2}$	$o_{u,3}$	70	270
$o_{u,3}$	$o_{u,4}$	0	0

## Distributed scheduler framework

**Fig. 4:**
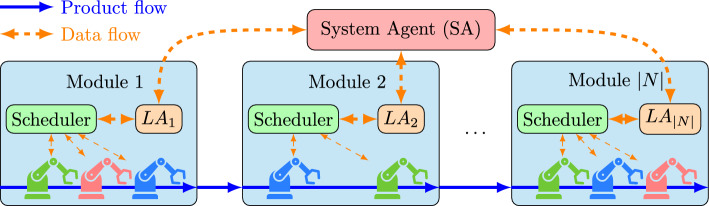
Production line assembly and agent architecture. The system agent (SA) interacts with the local agents (LAs) and the LAs interact with the module’s scheduler

In this section, we explain our method to generate a schedule for a modular, tightly coupled production line. We propose a MAS architecture (Sect. “Multi-agent system architecture”) that propagates constraints between modules until all modules have reached a consensus on mutually consistent schedules. Figure 4 shows an updated version of Fig. 1 with the added MAS coordinating between schedulers. We define the (system and local) agents and the information that they exchange to produce a schedule for a production line in Sects. “System agent” and “Local agent”. Section “Requirements on the LAs and local schedulers” discusses the requirements on the local schedulers and the local agents for the proposed approach to work. In Sect. “Proof of soundness”, we prove soundness of the approach. Section “Some concluding remarks” concludes.

### Multi-agent system architecture

Our MAS architecture is composed of a system agent (SA) and multiple local agents (LAs). Each local agent $LA_{i}$ is in charge of module $n_{i}$, contains the local problem $\tau _{i}$, and interacts with its scheduler. This architecture allows the modules to only share a small portion of their inner workings (for reasons of reusability and/or because they are produced by different manufacturers) by only sharing timing bounds on their input or output operations.

The LAs communicate with the local schedulers and model their space of feasible solutions by representing the relative start resp. completion times of the input and output operations of all pairs of jobs in the system with a lower and upper bound. The bounds are sent to the SA that forwards them to the other LAs in the system. Each $LA_{i}$ models the received bounds as constraints of their local re-entrant problem. Due to transfer-point constraints CT1 and CT2, output constraints of a module cannot be mapped directly to input constraints of the next one. Hence, since the SA knows all the transfer points, it translates the constraints between modules.

#### Example 5.1

**Fig. 5:**
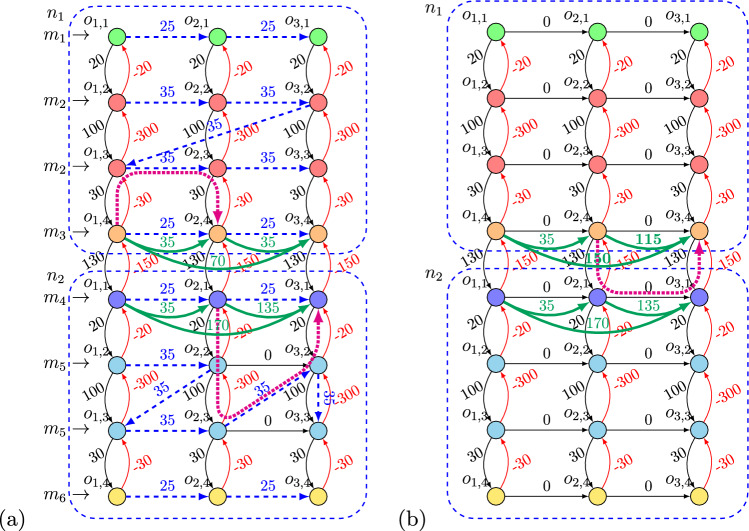
Propagation of constraints between modules $n_{1}$ and $n_{2}$ of Example 4.1. Fig. **a** shows an extension of Fig. 3 with the border constraints, computed by the LAs, drawn as arrows. The arrows show some of the longest paths used to compute the value of these constraints. Fig.** b** shows a constraint-graph result of propagating the constraints between modules $n_{1}$ and $n_{2}$. The graph is shown without the sequence edges for simplicity. Thus, now in module $n_{1}$ the value of the constraint from $o_{2,4}$ to $o_{3,4}$ is updated to 115 from the previous value 35 as a result of the longest path highlighted with the arrow. This propagation is done by the SA

Suppose we schedule Example 4.1 from Sect. “Production-line problem”. Figure 5 shows a general overview of the workings of the constraint propagation mechanism, of which the details are elaborated in the upcoming subsections. First, each LA collects constraints in the form of minimal setup times and due dates for its last resp. first operations resulting from the chosen local machine sequences. These bounds are included in the local constraint-graph representation of the scheduling problem at hand, as indicated in Fig. 5a ( arrows), and sent to the SA. Then, the SA translates and propagates these constraints. Thus, the bound between $o_{2,1}$ and $o_{3,1}$ with value 135 from module $n_{2}$, obtained from the length of the longest path highlighted by the arrow in Fig. 5a, is propagated to module $n_{1}$ as a bound between $o_{2,4}$ and $o_{3,4}$ with value 115. To do so, the SA computes the longest path in the transfer point. This path is highlighted by the arrow in Fig. 5b. $\blacksquare$

### System agent

The SA uses Algorithm 1 to try to find a consensus between the schedules of the LAs and forwards the necessary constraints such that each LA generates a schedule that, together, provide a globally feasible schedule.

**Algorithm 1:**
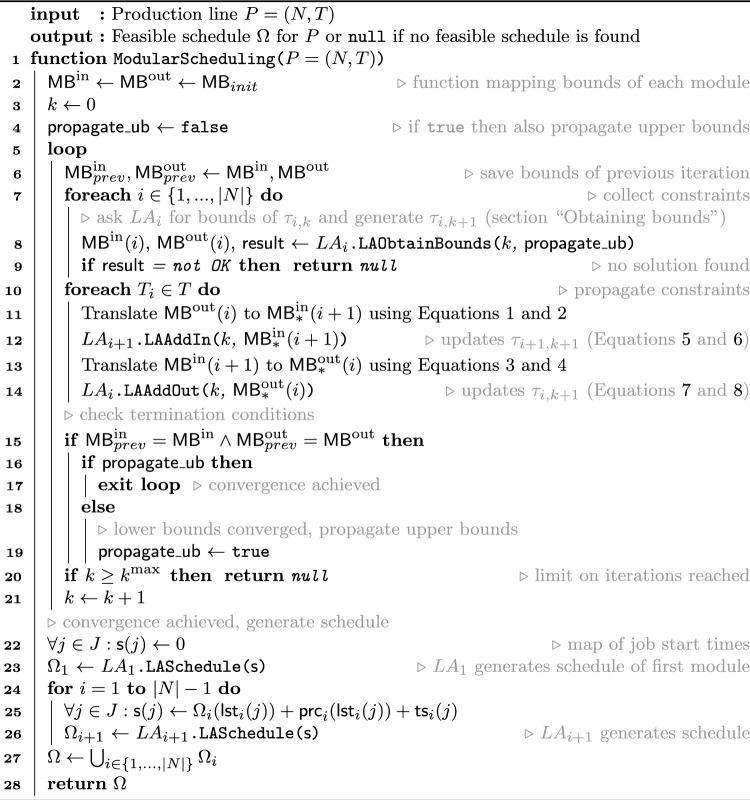
System agent

#### Finding consensus

To find a consensus between the LAs, the SA follows an iterative approach in lines 5 to 21. During this step, the LAs generate constraints that the SA stores in $\mathsf{MB}^{\textrm{in}}$ and $\mathsf{MB}^{\textrm{out}}$ (lines 7 to 9). After collecting all constraints, the SA translates and forwards them to the appropriate destination according to the defined transfer points (lines 10 to 14).    

At every iteration $k$, the LAs use the LAObtainBounds function of line 8 (described in Algorithm 2) to obtain the bounds abstracting the space of feasible solutions of their local problems $\tau _{i,k}$. Initially, the problem tuple is the one provided by the module, i.e., $\tau _{i,0} = \tau _{i}$. The local problem to be solved is updated in every iteration of the propagation step with the bounds received from neighbouring modules. The LAObtainBounds method provides a pair $(t^{\textrm{LB}}, t^{\textrm{UB}})$ with a lower and upper bound for each pair of jobs in the system. Section  “Constraints as bounds” precisely defines these bounds. The LA obtains the bounds by evaluating the sequences of operations per machine obtained from the local scheduler. The bounds correspond to the lengths of the longest paths between boundary operations in a constraint-graph representation of the local scheduling problem ( arrows in Fig. 5a). After producing the bounds, the LA also creates a new problem tuple $\tau _{i,k+1} = (J_{i}, M_{i}, \mathsf{ops}_{i}, \mathsf{mch}_{i}, \mathsf{prc}_{i},\mathsf{si}_{i,k+1},\mathsf{sd}_{i},\mathsf{dd}_{i,k+1})$ and adds the bounds into $\tau _{i,k+1}$ as constraints (Sect. “Incorporating constraints”, the arrows in the constraint-graph representation in Fig. 5a). This problem tuple will be updated in the constraint propagation phase with the constraints of other modules. The details are further explained in Sect. “Obtaining bounds”.

The bounds of input operations are collected in $\textsf{MB}^{\textrm{in}}$ and the bounds of output operations in $\mathsf{MB}^{\textrm{out}}$. In our initialisation step, lines 2 to 4, we define the $\mathsf{MB}_{init}$ function as a function returning the tuple $(0,\bot )$ for each module and pair of jobs from production line $P$. This captures the fact that, initially, no bounds are known on the relative input and output times of jobs in a module (the symbol $\bot$ represents the absence of an upper bound).    

There might be instances where a local scheduler fails to find a solution for a given problem. This could be due to the problem being unsolvable from the beginning or because the propagated bounds have shrunk the feasible solution space of the local problem to such an extent that no viable solutions are left. When this happens, the scheduling process is halted (line 9). A back-up solution should be considered for such cases. Depending on the local schedulers used, it may be possible to use back-up schedules (see Sect. “Experimental evaluation”); alternatively, some of the problem constraints can be relaxed.

After the constraints are collected, for each transfer point $T_{i}$, the SA propagates the constraints of the output operations of module $n_{i}$ to module $n_{i+1}$ (line 12) and propagates the constraints of input operations of module $n_{i+1}$ to $n_{i}$ (line 14). Because the constraints cannot be mapped directly to an adjacent module, due to transfer-point-constraints CT1 and CT2, the SA translates them to $\mathsf{MB}^{\textrm{in}}_{*}(i+1)$ and $\mathsf{MB}^{\textrm{out}}_{*}(i)$, respectively, before sending them (lines 11 and 13). This translation is explained in Sect. “Constraint propagation”. The extra constraints are included in the sequence-independent setup times, $\mathsf{si}_{i,k+1}$, and the due dates, $\mathsf{dd}_{i,k+1}$, respectively. This process is explained in Sect. “Incorporating constraints” and illustrated in Fig. 5b (the arrows).

This iterative process stops when convergence is achieved. That is, when all the bounds $\mathsf{MB}^{\textrm{in}}$ and $\mathsf{MB}^{\textrm{out}}$ are the same for two consecutive iterations (line 17). This means that the modules have reached a consensus on their constraints and that the constraint requirements of one module match the constraint requirements of its neighbours for each module in the system. To avoid reducing the space of feasible schedules of modules too fast, Algorithm 1 defers the propagation of upper bounds. Until line 19 is reached, only lower bounds are being propagated. Function LAObtainBounds only outputs lower bounds when propagate_ub is false (see Sect. “Obtaining bounds”). After line 19 is reached, propagate_ub is true and upper bounds are propagated.

In some cases, if the local schedulers repeatedly alternate between the same schedules, the modules do not reach a consensus. This problem cannot be avoided without imposing additional assumptions on the local schedulers, as explained in Sect. “Termination analysis”. That is why there is a limit of $k^{\max {}}$ iterations in line 20. When this limit is reached, Algorithm 1 terminates without providing a schedule.

##### Example 5.2

Continuing Example 5.1, assume that the local schedulers always choose the same sequences. Table 3 shows the values of the bounds that the SA receives, translates and propagates across iterations. $\mathsf{MB}^{\textrm{in}}(1)$ and $\mathsf{MB}^{\textrm{out}}(2)$ are omitted because modules $n_{1}$ and $n_{2}$ do not have a predecessor and successor respectively.

In Iteration 1, only lower bounds are propagated. Thus, the lower diagonal parts of the boxes in Table 3 are empty. $LA_{2}$ obtains the value ${\color{red}{135}}$ from the length of the longest path in Fig. 5a. The SA translates the value to ${\color{red}{115}}$ by finding the length of the longest path from $o_{2,4}$ to $o_{3,4}$ in the transfer point ( in Fig. 5b), see Sect. “Constraint propagation”. In Iteration 2, module $n_{1}$ has incorporated the constraints of $\mathsf{MB}^{\textrm{out}}_{*}(1)$ of the previous iteration so that the bounds that it sends to the SA satisfy these constraints. That is why the bound from job $j_{2}$ to $j_{3}$ in $\mathsf{MB}^{\textrm{out}}(1)$ in the second iteration is 115.    

Iteration 3 reaches lower bounds convergence and hence is identical to Iteration 2; it is omitted from Table 3. The SA continues iterating using also upper bounds (setting propagate_ub to true in the algorithm). The upper bounds are represented as negative values starting from Iteration 4. In Iteration 5, module $n_{2}$ has incorporated the upper bounds of module $n_{1}$ and its schedule satisfies them. Finally, in Iteration 6, not shown in Table 3, all LAs produce the same bounds as in Iteration 5 and consensus has been reached. The schedule generation that then follows is explained in the running example in Sect. “Production-line schedule generation”. $\blacksquare$

**Table 3:** Values of the bounds and their translation across iterations of the running example

To		$\mathsf{MB}^{\textrm{out}}(1)$			$\mathsf{MB}^{\textrm{in}}_{*}(2)$			$\mathsf{MB}^{\textrm{in}}(2)$			$\textsf{MB}^{\textrm{out}}_{*}(1)$		
From		$j_{1}$	$j_{2}$	$j_{3}$	$j_{1}$	$j_{2}$	$j_{3}$	$j_{1}$	$j_{2}$	$j_{3}$	$j_{1}$	$j_{2}$	$j_{3}$
Iter. 1	$j_{1}$	0	35	70	0	15	50	0	35	170	0	15	150
	$j_{2}$		0	35		0	15		0	${\color{red}{135}}$		0	${\color{red}{115}}$
	$j_{3}$			0			0			0			0
Iter. 2	$j_{1}$	0	35	**150**	0	15	**130**	0	35	170	0	15	150
	$j_{2}$		0	**115**		0	**95**		0	135		0	115
	$j_{3}$			0			0			0			0
Iter. 4	$j_{1}$	0	35	150	0	15	130	0	35	170	0	15	150
	$j_{2}$	**−150**	0	115	**− 170**	0	95	**− 265**	0	135	**− 285**	0	115
	$j_{3}$	**− 265**	**− 230**	0	**− 285**	**− 250**	0			0			0
Iter. 5	$j_{1}$	0	35	150	0	15	130	0	35	170	0	15	150
	$j_{2}$	−150	0	115	− 170	0	95	**−150**	0	135	**− 170**	0	115
	$j_{3}$	− 265	− 230	0	− 285	− 250	0	**− 285**	**-250**	0	**−305**	**− 270**	0

Changes between iterations are highlighted in bold face. Each value from $j_{u}$ to $j_{v}$ with $u < v$ represents a lower bound while each negative value from $j_{v}$ to $j_{u}$ represents an upper bound (i.e., as a negative lower bound in the opposite direction). Each row represents a single iteration. Repeated iterations (3 and 6) have been omitted. The red highlighted entries are discussed in Example 5.2

Algorithm 1 detects convergence by comparing the obtained bounds in two consecutive iterations. This can be implemented more efficiently by evaluating the bounds ($\mathsf{MB}^{\textrm{in}}$, $\mathsf{MB}^{\textrm{out}}$) that the modules send and checking whether the translated bounds ($\mathsf{MB}^{\textrm{in}}_{*}$, $\mathsf{MB}^{\textrm{out}}_{*}$) would really update the local problems of the modules. If, for instance, the bounds $\mathsf{MB}(i)$ that a module $n_{i}$ sends are “stronger” than the bounds $\mathsf{MB}_{*}(i)$ that will be incorporated in the module, then the local problem will not be affected. That is, if, for all pairs of jobs, all the lower bounds of $\mathsf{MB}(i)$ are at least equal to the lower bounds of $\mathsf{MB}_{*}(i)$ and the upper bounds of $\mathsf{MB}(i)$ are at most equal to the upper bounds of $\mathsf{MB}_{*}(i)$, then convergence has occurred. In the above example, for instance, this means that already in Iteration 2, it is possible to detect convergence of lower bounds, avoiding the explicit computation of Iteration 3.      

#### Production-line schedule generation

If a consensus between modules is found, then the input and/or output constraints of a module satisfy the constraints of its neighbours for each module in the system. Thus, it is then possible to generate a feasible schedule for the production line. We generate a schedule in a distributed manner in lines 22–27 of Algorithm 1 by iteratively providing the start times of the input operations of a module (Lines 23 and 26) and obtaining the completion times of its output operations.

##### Definition 1

*(ASAP schedule)* We define the as-soon-as-possible (ASAP) schedule with the lower bounds function ${\textsf{s}} : J \hookrightarrow \mathbb {R}$ for a flow shop $\tau$ and the sequences $\mathsf{seq}$ as a feasible schedule $\Omega$ where $\Omega (\mathsf{fst}(j)) \ge {\mathsf{s}}(j)$ for all $j$ in the domain of ${\mathsf{s}}$, where the start times of the operations of each machine $m$ in $M$ follow the order of the sequence $\mathsf{seq}(m)$ (i.e., $\Omega (\mathsf{seq}(m)(i)) < \Omega (\mathsf{seq}(m)(j))$ if and only if $i < j$), and where $\Omega (o)$ is minimal for all $o$ in $O$.    

In lines 23 and 26, the LASchedule method of the LAs (defined in Algorithm 5 of Sect. “Schedule generation”) is called. It generates the ASAP schedule from the lower bounds function ${\mathsf{s}}$ and the sequences $\mathsf{seq}_{i}$ that the local schedulers used to reach consensus. The ASAP schedule is required for the global schedule to be valid, as elaborated in Sect. “Proof of soundness”. The values of the lower bounds function ${\mathsf{s}}$ for each next module are determined by adding the transfer times of the output operations of the previous module to the scheduled completion time of these output operations. This ensures that a job in $n_{i+1}$ cannot start until it is finished in module $n_{i}$ and has arrived at module $n_{i+1}$. We prove that the global schedule generated in these steps is feasible in Sect. “Proof of soundness”. We provide the precise requirements for the local schedulers in Sect. “Requirements on the LAs and local schedulers”. Note that, even though line 25 refers to start and processing times of an operation, in a practical implementation, it may not be feasible or desirable that operation processing times and/or start times of internal operations of modules are made available to the SA. In such a case, it suffices to communicate only completion times of the local schedules from the LAs to the SA. Algorithm 1 provides a global schedule containing all operations so we may reason about feasibility of this global schedule.    

##### ***Example 5.3***

**Fig. 6:**
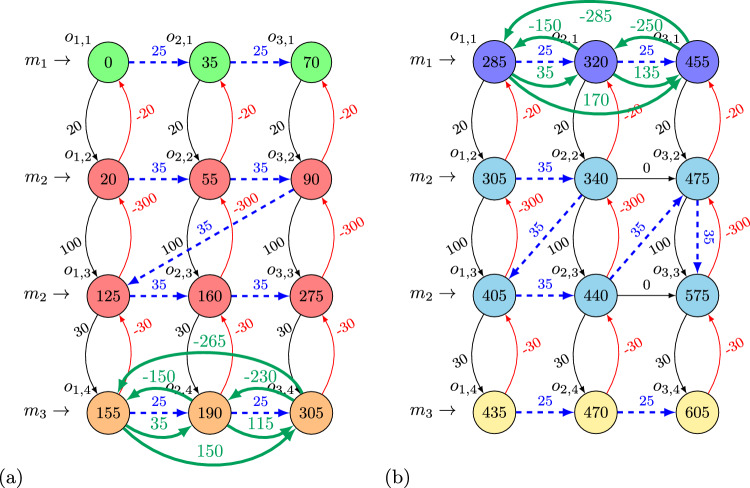
Constraint graph of modules $n_{1}$ (**a**) and $n_{2}$ (**b**) of the running example with schedule and final bounds ( arrows). The value inside each node is the start time of that operation according to global schedule $\Omega$. Schedule $\Omega$ is built from local schedules $\Omega _{1}$ and $\Omega _{2}$ that are globally feasible in the production line defined in Example 4.1

Recall Example 5.2. After Iteration 6, the SA asks $LA_{1}$ to generate a schedule. Figure 6a shows a constraint graph with schedule $\Omega _{1}$. The SA receives the output values ($155$, $190$, and $305$) of $\Omega _{1}$, adds the minimum transport times ($130$), and passes these values ($285$, $320$, and $435$) to $LA_{2}$ as lower bounds on the start times of its first operations. $LA_{2}$ generates the ASAP schedule $\Omega _{2}$ using these values as lower bounds and produces the schedule of Fig. 6b. Notice how the start time of operation $o_{3,1}$, $455$, is greater than the received lower bound $435$, but that this value still satisfies the transfer due date constraint of $150$ (see Fig. 3). $\blacksquare$

#### Constraints as bounds

The SA and the LAs exchange bounds as an abstraction of the local problem constraints. We first formally define them in Definition 3 and then explain how the SA gathers them. Section “Obtaining bounds” explains how an LA obtains these bounds from a local problem tuple.

We model the space of feasible solutions of a module by representing, for each pair of jobs $j_{u}$ and $j_{v}$ with $u < v$, the lower and upper bound on the difference between the start times of two input operations and on the difference between completion times of two output operations. Each local agent $LA_{i}$ asks the local scheduler to generate the sequences of operations $\mathsf{seq}_{i}$ for the machines of the module and produces the bounds by evaluating these sequences.

##### Definition 2

*(Feasible schedules given machine sequences)* We define $S_{\mathsf{seq}_{i,k}}$ as the set of all feasible schedules $\Omega _{i,k}$ of problem $\tau _{i,k}$ at iteration $k$ that have the machine sequences $\mathsf{seq}_{i,k}$.

##### Definition 3

*(Bounds)* For the input operations of $$\tau _{i,k}$$, we define $$\boldsymbol{t_{i,k,u,v}^{\mathrm{in},\mathrm{LB}}}$$ and $$\boldsymbol{t_{i,k,u,v}^{\mathrm{in},\mathrm{UB}}}$$ as the minimum and maximum difference $$\Omega _{{i,k}} (\mathsf{fst}_{i} (j_{v} ))$$$$- \Omega _{{i,k}} (\mathsf{fst}_{i} (j_{u} )) $$, respectively, over all $$\Omega _{i,k}$$ in $$S_{\mathsf{seq}_{i,k}}$$, and, for the output operations, we define $$\boldsymbol{t_{i,k,u,v}^{\mathrm{out},\mathrm{LB}}}$$ and $$\boldsymbol{t_{i,k,u,v}^{\mathrm{out},\mathrm{UB}}}$$ as the minimum and maximum difference $$\Omega _{{i,k}} (\mathsf{lst}_{i} (j_{v} )) $$$${} + \mathsf{prc}_{i} (\mathsf{lst}_{i} (j_{v} )) $$$${} - \big( \Omega _{{i,k}} (\mathsf{lst}_{i} (j_{u} )){}$$$$+\mathsf{prc}_{i}(\mathsf{lst}_{i}(j_u))\big)$$, respectively, over all $$\Omega _{i,k}$$ in $$S_{\mathsf{seq}_{i,k}}$$.    

It is possible that the upper bounds $t_{i,k,u,v}^{\textrm{in},\textrm{UB}}$ or $t_{i,k,u,v}^{\textrm{out},\textrm{UB}}$ do not exist, as it may be possible for operations to be delayed indefinitely. For simplicity, in the remainder, we drop super- and subscripts that are not relevant or can be deduced from the context.

Because the SA and the LAs exchange bounds for all pairs of jobs, we define the mapping function $B : \{u \in \mathbb {N} \mid 1 \le u \le | J | \}^{2} \hookrightarrow \left( \mathbb {R} \cup \{\bot \} \right) ^{2}$ that returns a tuple $(t_{u,v}^{\textrm{LB}}, t_{u,v}^{\textrm{UB}})$ representing the value of the lower ($t_{u,v}^{\textrm{LB}}$) and upper ($t_{u,v}^{\textrm{UB}}$) bound for each pair of jobs $j_{u}$ and $j_{v}$ with $u < v$. Each element of the returned tuple can either be a real value or the symbol $\bot$ meaning the absence of a bound. For consistency, we require the second element of the pair to be at least the value of the first if both exist. So, we have, for instance, that $B(u, v) = (t_{i,k,u,v}^{\textrm{in},\textrm{LB}}, t_{i,k,u,v}^{\textrm{in},\textrm{UB}})$. We define the mappings $\mathsf{MB}^{\textrm{in}}$ and $\mathsf{MB}^{\textrm{out}}$ used in Algorithm 1 as functions with domain $\{i \in \mathbb {N} \mid 1 \le i \le |N|\}$ mapping a module index to its mapping of bounds (i.e., a function $B$ as defined above); $\mathsf{MB}^{\textrm{in}}(i)$ and $\mathsf{MB}^{\textrm{out}}(i)$ contain all the input and output bounds for all job pairs of module $n_{i}$, respectively. Recall Example 5.2 that illustrates the use of these mappings in the iterative consensus process.        

#### Constraint propagation

The maps of bounds $B^{\textrm{in}}$ and $B^{\textrm{out}}$ generated by the local agents in each iteration of the main loop of Algorithm 1 are collected by the SA in the mappings $\mathsf{MB}^{\textrm{in}}$ and $\mathsf{MB}^{\textrm{out}}$, respectively. Since they represent constraints of the sending module, the SA translates them to valid constraints of the module that receives them. For the transfer point $T_{i}$, the SA translates the maps $\mathsf{MB}^{\textrm{out}}(i)$ and $\mathsf{MB}^{\textrm{in}}(i+1)$ into the maps $\mathsf{MB}^{\textrm{in}}_{*}(i+1)$ and $\mathsf{MB}^{\textrm{out}}_{*}(i)$, respectively. $LA_{i}$ and $LA_{i+1}$ then receive $\mathsf{MB}^{\textrm{out}}_{*}(i)$ and $\mathsf{MB}^{\textrm{in}}_{*}(i+1)$, respectively, which they use to update the problems $\tau _{i,k+1}$ and $\tau _{i+1,k+1}$.        

**Fig. 7:**
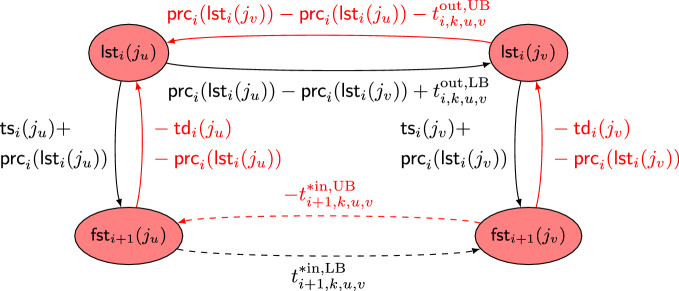
All possible constraints between jobs $j_{u}$ and $j_{v}$ at transfer point $T_{i}$

The translation process accounts for all the constraints of the transfer point $T_{i}$. Figure 7 shows a constraint graph of the possible transfer-point constraints between two jobs $j_{u}$ and $j_{v}$ of two consecutive modules $n_{i}$ and $n_{i+1}$. The bounds $\mathsf{MB}^{\textrm{out}}(i)(u,v) = \big (t_{i,k,u,v}^{\textrm{out},\textrm{LB}}, t_{i,k,u,v}^{\textrm{out},\textrm{UB}}\big )$ are translated into $\mathsf{MB}^{\textrm{in}}_{*}(i+1)(u,v)=\big (t_{i+1,k,u,v}^{*\textrm{in},\textrm{LB}}, t_{i+1,k,u,v}^{*\textrm{in},\textrm{UB}}\big )$ and $\mathsf{MB}^{\textrm{in}}(i+1)(u,v) = \big (t_{i+1,k,u,v}^{\textrm{in},\textrm{LB}},t_{i+1,k,u,v}^{\textrm{in},\textrm{UB}}\big )$ into $\mathsf{MB}^{\textrm{out}}_{*}(i)(u,v) = \big (t_{i,k,u,v}^{*\textrm{out},\textrm{LB}}, t_{i,k,u,v}^{*\textrm{out},\textrm{UB}}\big )$. If the upper bounds $t_{i+1,k,u,v}^{\textrm{in},\textrm{UB}}$ or $t_{i,k,u,v}^{\textrm{out},\textrm{UB}}$ do not exist, they are not propagated.

$t_{i+1,k,u,v}^{*\textrm{in},\textrm{LB}}$ is the propagation of the output constraint $t_{i,k,u,v}^{\textrm{out},\textrm{LB}}$ of module $n_{i}$ in iteration $k$ when applied to the input of module $n_{i+1}$ in iteration $k+1$, accounting for the transfer constraints. In the graph from Fig. 7, $t_{i,k,u,v}^{\textrm{out},\textrm{LB}}$ is represented as part of the weight of the edge from $\mathsf{lst}_{i}(j_{u})$ to $\mathsf{lst}_{i}(j_{v})$. Because $t_{i,k,u,v}^{\textrm{out},\textrm{LB}}$ is a constraint on the relative completion time between the operations, whereas the edges in a constraint graph represent constraints on start times, the constraint in the graph is corrected with the processing times $\mathsf{prc}_{i}(\mathsf{lst}_{i}(j_{v}))$ and $\mathsf{prc}_{i}(\mathsf{lst}_{i}(j_{u}))$. Bound $t_{i+1,k,u,v}^{*\textrm{in},\textrm{LB}}$ is represented by the dashed edge from $\mathsf{fst}_{i+1}(j_{u})$ to $\mathsf{fst}_{i+1}(j_{v})$. The latter is to be added to $\tau _{i+1,k+1}$. To properly model all the constraints of the transfer point, the value of this edge needs to be equal to the length of the longest path that goes from $\mathsf{fst}_{i+1}(j_{u})$ to $\mathsf{fst}_{i+1}(j_{v})$. This path is the sequence of nodes $\langle \mathsf{fst}_{i+1}(j_{u}), \mathsf{lst}_{i}(j_{u}), \mathsf{lst}_{i}(j_{v}), \mathsf{fst}_{i+1}(j_{v})\rangle$. Putting this together, we obtain:

1 $$\begin{aligned} \begin{aligned} t_{i+1,k,u,v}^{*\textrm{in},\textrm{LB}}&= -\mathsf{td}_{i}(j_{u}) -\mathsf{prc}_{i}(\mathsf{lst}_{i}(j_{u})) + \mathsf{prc}_{i}(\mathsf{lst}_{i}(j_{u})) -\mathsf{prc}_{i}(\mathsf{lst}_{i}(j_{v})) \\&\quad + t_{i,k,u,v}^{\textrm{out},\textrm{LB}} + \mathsf{prc}_{i}(\mathsf{lst}_{i}(j_{v})) + \mathsf{ts}_{i}(j_{v}) \\&= -\mathsf{td}_{i}(j_{u}) + \mathsf{ts}_{i}(j_{v}) + t_{i,k,u,v}^{\textrm{out},\textrm{LB}} \end{aligned} \end{aligned}$$

For $t_{i+1,k,u,v}^{*\textrm{in},\textrm{UB}}$, we use the path that goes from $\mathsf{fst}_{i+1}(j_{v})$ to $\mathsf{fst}_{i+1}(j_{u})$ with negated length. We use the same method for the output bounds from $\mathsf{MB}^{\textrm{in}}(i+1)(u,v) = \big (t_{i+1,k,u,v}^{\textrm{in},\textrm{LB}}, t_{i+1,k,u,v}^{\textrm{in},\textrm{UB}}\big )$ to $\mathsf{MB}^{\textrm{out}}_{*}(i)(u,v) = \big (t_{i,k,u,v}^{*\textrm{out},\textrm{LB}},t_{i,k,u,v}^{*\textrm{out},\textrm{UB}}\big )$. As a result, we obtain:

2 $$\begin{aligned} t_{i+1,k,u,v}^{*\textrm{in},\textrm{UB}}&= -\mathsf{ts}_{i}(j_{u}) + t_{i,k,u,v}^{\textrm{out},\textrm{UB}} + \mathsf{td}_{i}(j_{v}) \end{aligned}$$

3 $$\begin{aligned} t_{i,k,u,v}^{*\textrm{out},\textrm{LB}}&= \mathsf{ts}_{i}(j_{u}) + t_{i+1,k,u,v}^{\textrm{in},\textrm{LB}} - \mathsf{td}_{i}(j_{v}) \end{aligned}$$

4 $$\begin{aligned} t_{i,k,u,v}^{*\textrm{out},\textrm{UB}}&= -\mathsf{ts}_{i}(j_{v}) + t_{i+1,k,u,v}^{\textrm{in},\textrm{UB}} + \mathsf{td}_{i}(j_{u}) \end{aligned}$$

      

##### Example 5.4

Recall Example 5.1. Figure 5b illustrates how the bound of module $n_{1}$ between operations $o_{2,4}$ and $o_{3,4}$ is obtained. Recall that $t_{i,k,u,v}$ represents a bound from the first/last operation of $j_{u}$ to the first/last operation of $j_{v}$ of module $n_{i}$ at iteration $k$. The superscript “$\textrm{in}$” denotes input bounds (first operations of jobs), “$\textrm{out}$” denotes output bounds (last operations of jobs), “$\textrm{LB}$” denotes lower bounds and “$\textrm{UB}$” denotes upper bounds. $LA_{2}$ produces the bound $t_{2,1,2,3}^{\textrm{in},\textrm{LB}} = 135$ at iteration $k = 1$ ($\mathsf{MB}^{\textrm{in}}(2)(2,3)$ in Example 5.2, highlighted in red in Table 3). To obtain the translated output lower bound of module $n_{2}$, $t_{1,1,2,3}^{*\textrm{out},\textrm{LB}}$, we use Equation 3. The transfer setup time is $\mathsf{ts}_{1}(j_{2}) = 110$ and the due date is $\mathsf{td}_{1}(j_{3}) = 130$. Putting it all together gives

$$\begin{aligned} t_{1,1,2,3}^{*\textrm{out},\textrm{LB}} = \underbrace{110}_{\mathsf{ts}_{1}(j_{2})} + \underbrace{135}_{t_{2,1,2,3}^{\textrm{in},\textrm{LB}}} - \underbrace{130}_{\mathsf{td}_{1}(j_{3})} = 115 \end{aligned}$$

as a translated output lower bound. $\blacksquare$

### Local agent

The LA bridges the local scheduler and the SA to produce a globally feasible schedule. To do so, it (i) produces bounds abstracting the local schedules (LAObtainBounds, Sect. “Obtaining bounds”), (ii) consolidates internal bounds and bounds received from the SA as constraints in the local problem (LAAddIn, LAAddout, Sect. “Incorporating constraints”), and (iii) generates the local schedule and forwards the times of output operations (LASchedule, Sect. “Schedule generation”).

#### Obtaining bounds

The LA is responsible for producing the bounds of Definition 3 needed by the SA. The LA uses Algorithm 2 to compute these bounds at every iteration $k$. Initially, it requests the local scheduler to generate the sequences of operations per machine (line 2). It then generates a constraint graph from these sequences (line 4) and evaluates the sequences (lines 5 to 17) to produce the bounds. In our implementation, we reuse the graph across iterations, updating only the constraints that have changed. For simplicity of presentation, Algorithm 2 derives the full graph for each iteration.

**Algorithm 2:**
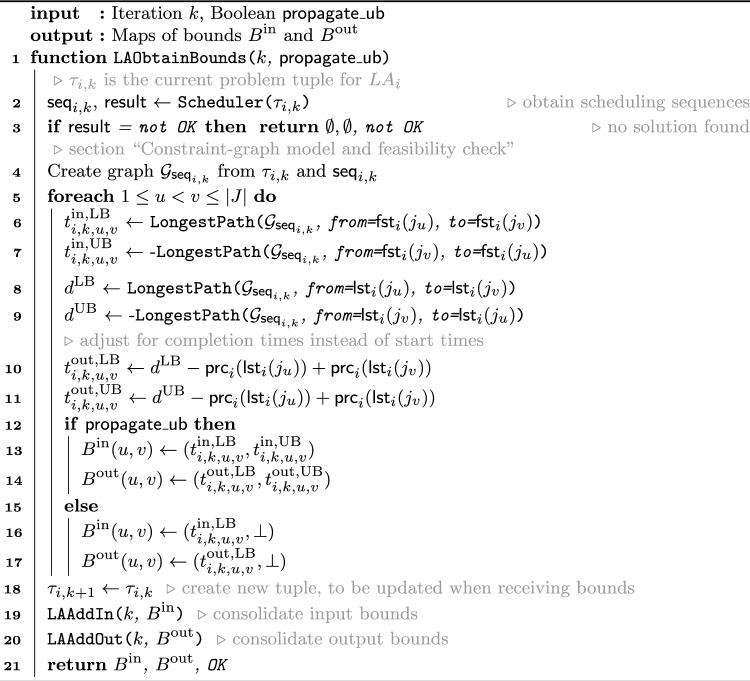
Obtaining $$t_{i,k,u,v}^{\textrm{in},\textrm{LB}}$$, $$t_{i,k,u,v}^{\textrm{in},\textrm{UB}}$$, $$t_{i,k,u,v}^{\textrm{out},\textrm{LB}}$$, $$t_{i,k,u,v}^{\textrm{out},\textrm{UB}}$$

Because the lower and upper bounds $t_{i,k}^{\textrm{LB}}$ and $t_{i,k}^{\textrm{UB}}$ are related to the minimum or maximum delays between two operations, the values of these bounds are obtained by evaluating the longest path between the operations that the bounds represent. The LongestPath function is used to compute the longest path from the node from to the node to in the graph passed as the first parameter. The value of $t_{i,k,u,v}^{\textrm{in},\textrm{LB}}$ is the length of the longest path that goes from $\mathsf{fst}_{i}(j_{u})$ to $\mathsf{fst}_{i}(j_{v})$ (line 6). This length is the same as the minimum value of $\Omega _{i,k}(\mathsf{fst}_{i}(j_{v})) - \Omega _{i,k}(\mathsf{fst}_{i}(j_{u}))$ for all schedules $\Omega _{i,k}$ in $S_{\mathsf{seq}_{i,k}}$. The value of bound $t_{i,k,u,v}^{\textrm{in},\textrm{UB}}$ is determined in a similar way (line 7). The path with length $w$ from $\mathsf{fst}_{i}(j_{v})$ to $\mathsf{fst}_{i}(j_{u})$ imposes the constraint $\Omega _{i,k}(\mathsf{fst}_{i}(j_{v})) + w \le \Omega _{i,k}(\mathsf{fst}_{i}(j_{u}))$. Since $t_{i,k,u,v}^{\textrm{in},\textrm{UB}}$ represents the constraint $\Omega _{i,k}(\mathsf{fst}_{i}(j_{u})) + t_{i,k,u,v}^{\textrm{in},\textrm{UB}} \ge \Omega _{i,k}(\mathsf{fst}_{i}(j_{v}))$, the value of $t_{i,k,u,v}^{\textrm{in},\textrm{UB}}$ is $-w$. Similarly, the values for $t_{i,k,u,v}^{\textrm{out},\textrm{LB}}$ and $t_{i,k,u,v}^{\textrm{out},\textrm{UB}}$ are computed from longest paths in the constraint graph in Lines 8, 9, respectively. But, because these bounds are defined in terms of completion times instead of start times, the computed values are updated in Lines 10, 11. If the constraint graph has no path for a given bound, LongestPath returns the symbol $\bot$ representing the absence of a bound.

Algorithm 2 only returns the upper bounds if propagate_ub is true. Otherwise, it returns $\bot$ as value for the upper bounds. Thus, in lines 12 to 17 the right value is set. This is used in Algorithm 1 to avoid reducing the space of feasible solutions too fast. It is further explained in Sect. “Finding consensus”.

In line 18, the LA generates the new problem tuple $\tau _{i,k+1}$ that will be updated with the bounds from its neighbours. As mentioned, our implementation maintains a constraint graph that is incrementally updated in every iteration. The explicit problem tuple update is included to clarify that the local scheduling problem to be solved changes in every iteration and to support the soundness proof later on.

Finally, to expedite convergence, the LA incorporates the bounds that it transmits to the SA as constraints for the problem tuple of the next iteration $\tau _{i,k+1}$ in lines 19 to 20. This method accelerates convergence as the bounds are disseminated to the neighbours during the propagation phase, and in turn, the neighbours send these values (or tighter constraints) back in their propagation step of the next iterations.

##### Example 5.5

As already introduced, in Fig. 5a, the arrow of module $n_{1}$ is the longest path used to compute the value of the bound, $t_{1,1,1,2}^{\textrm{out},\textrm{LB}}$, from $o_{1,4}$ to $o_{2,4}$ in iteration 1. The paths of the other bounds of module $n_{1}$ are not shown. Similarly, for module $n_{2}$, the arrow is the longest path used to compute the value of the bound $t_{2,1,2,3}^{\textrm{in},\textrm{LB}}$ from $o_{2,1}$ to $o_{3,1}$. $\blacksquare$

#### Incorporating constraints

$LA_{i}$ receives the maps $\mathsf{MB}^{\textrm{in}}_{*}(i)$ and $\mathsf{MB}^{\textrm{out}}_{*}(i)$ from the SA (lines 10 to 14 of Algorithm 1). The LA uses the constraints of $\mathsf{MB}^{\textrm{in}}_{*}(i)$ and $\mathsf{MB}^{\textrm{out}}_{*}(i)$ to update the problem $\tau _{i,k+1}$ derived from the problem of the current iteration $\tau _{i,k}$. The bounds $t_{i,k,u,v}^{*\textrm{in},\textrm{LB}}$ and $t_{i,k,u,v}^{*\textrm{in},\textrm{UB}}$ in the range of $\mathsf{MB}^{\textrm{in}}_{*}(i)$ and the bounds $t_{i,k,u,v}^{*\textrm{out},\textrm{LB}}$ and $t_{i,k,u,v}^{*\textrm{out},\textrm{UB}}$ in the range of $\mathsf{MB}^{\textrm{out}}_{*}(i)$ are added as constraints in $\tau _{i,k+1}$. As explained earlier, $LA_{i}$ incorporates the internal bounds that it generated already in Algorithm 2.

For every input lower bound $t_{i,u,v}^{\textrm{in},\textrm{LB}}$ either internal $\big (t_{i,k,u,v}^{\textrm{in},\textrm{LB}}\big )$ or external $\big (t_{i,k,u,v}^{*\textrm{in},\textrm{LB}}\big )$, i.e., $t_{i,u,v}^{\textrm{in},\textrm{LB}}$ in $\big \{t_{i,k,u,v}^{*\textrm{in},\textrm{LB}},t_{i,k,u,v}^{\textrm{in},\textrm{LB}}\big \}$, the LA includes the constraint $\Omega _{i,k+1}(\mathsf{fst}_{i}(j_{u})) + t_{i,u,v}^{\textrm{in},\textrm{LB}} \le \Omega _{i,k+1}(\mathsf{fst}_{i}(j_{v}))$ as a setup constraint $\mathsf{si}_{i,k+1}$ between operations $\mathsf{fst}_{i}(j_{u})$ and $\mathsf{fst}_{i}(j_{v})$. For every input upper bound $t_{i,u,v}^{\textrm{in},\textrm{UB}}$ with $t_{i,u,v}^{\textrm{in},\textrm{UB}}$ in $\big \{t_{i,k,u,v}^{*\textrm{in},\textrm{UB}}, t_{i,k,u,v}^{\textrm{in},\textrm{UB}}\big \}$, the LA includes the constraint $\Omega _{i,k+1}(\mathsf{fst}_{i}(j_{u})) + t_{i,u,v}^{\textrm{in},\textrm{UB}} \ge \Omega _{i,k+1}(\mathsf{fst}_{i}(j_{v}))$ as a due date constraint $\mathsf{dd}_{i,k+1}$ between operations $\mathsf{fst}_{i}(j_{u})$ and $\mathsf{fst}_{i}(j_{v})$. Similarly, for output lower bounds $t_{i,u,v}^{\textrm{out},\textrm{LB}}$ in $\big \{t_{i,k,u,v}^{*\textrm{out},\textrm{LB}}, t_{i,k,u,v}^{\textrm{out},\textrm{LB}}\big \}$, and $t_{i,u,v}^{\textrm{out},\textrm{UB}}$ in $\big \{t_{i,k,u,v}^{*\textrm{out},\textrm{UB}}, t_{i,k,u,v}^{\textrm{out},\textrm{UB}}\big \}$, the LA includes the constraints $\Omega _{i,k+1}(\mathsf{lst}_{i}(j_{u})) + t_{i,u,v}^{\textrm{out},\textrm{LB}} \le \Omega _{i,k+1}(\mathsf{lst}_{i}(j_{v}))$ and $\Omega _{i,k+1}(\mathsf{lst}_{i}(j_{u})) + t_{i,u,v}^{\textrm{out},\textrm{UB}} \ge \Omega _{i,k+1}(\mathsf{lst}_{i}(j_{v}))$ as a setup constraint $\mathsf{si}_{i,k+1}$ and a due date constraint $\mathsf{dd}_{i,k+1}$ respectively between operations $\mathsf{lst}_{i}(j_{u})$ and $\mathsf{lst}_{i}(j_{v})$. If a setup constraint $\mathsf{si}_{i,k+1}$ already exists, the value is updated with the maximum among the previous one and the new one and if a deadline $\mathsf{dd}_{i,k+1}$ already exists, the value is updated with the minimum among the previous value and the new one. Note that the received bounds need to be adjusted with the processing time of $\mathsf{fst}_{i}(j_{u})$. The constraints are added as follows^2^:

5 $$\begin{aligned} \mathsf{si}_{{i,k + 1}} & (\mathsf{fst}_{i} (j_{u} ),\mathsf{fst}_{i} (j_{v} )) =\\&\max \left( t_{{i,u,v}}^{{{\mathrm{in}},{\mathrm{LB}}}} - \mathsf{prc}_{i} (\mathsf{fst}_{i} (j_{u} )) , \mathsf{si}_{{i,k + 1}} (\mathsf{fst}_{i} (j_{u} ),\mathsf{fst}_{i} (j_{v} )) \right) \end{aligned}$$

6 $$\begin{aligned} \mathsf{dd}_{{i,k + 1}}& (\mathsf{fst}_{i} (j_{u} ),\mathsf{fst}_{i} (j_{v} )) = \\& \min \left( t_{{i,u,v}}^{{{\mathrm{in}},{\mathrm{UB}}}} - \,\mathsf{prc}_{i} (\mathsf{fst}_{i} (j_{u} )) ,\mathsf{dd}_{{i,k + 1}} (\mathsf{fst}_{i} (j_{u} ),\mathsf{fst}_{i} (j_{v} )) \right) \end{aligned}$$

To add the constraints implied by the bounds $\mathsf{MB}^{\textrm{out}}_{*}(i)(u,v) = \big (t_{i,k,u,v}^{*\textrm{out},\textrm{LB}}, t_{i,k,u,v}^{*\textrm{out},\textrm{UB}}\big )$ for the output operations, we use similar equations but account for the fact that the bounds relate to completion times of the last operations of jobs $j_{u}$ and $j_{v}$ instead:

7 $$\begin{aligned}\mathsf{si}_{{i,k + 1}} & (\mathsf{lst}_{i} (j_{u} ),\mathsf{lst}_{i} (j_{v} )) = \\& \max \left( t_{{i,u,v}}^{{{\mathrm{out}},{\mathrm{LB}}}} - \mathsf{prc}_{i} (\mathsf{lst}_{i} (j_{v} )) ,\mathsf{si}_{{i,k + 1}} (\mathsf{lst}_{i} (j_{u} ),\mathsf{lst}_{i} (j_{v} )) \right) \end{aligned}$$

8 $$\begin{aligned}\mathsf{dd}_{{i,k + 1}} &(\mathsf{lst}_{i} (j_{u} ),\mathsf{lst}_{i} (j_{v} )) = \\& \min \left( t_{{i,u,v}}^{{{\mathrm{out}},{\mathrm{UB}}}} - \mathsf{prc}_{i} (\mathsf{lst}_{i} (j_{v} )), \mathsf{dd}_{{i,k + 1}} (\mathsf{lst}_{i} (j_{u} ), \mathsf{lst}_{i} (j_{v} )) \right) \end{aligned}$$

    

**Algorithm 3:**
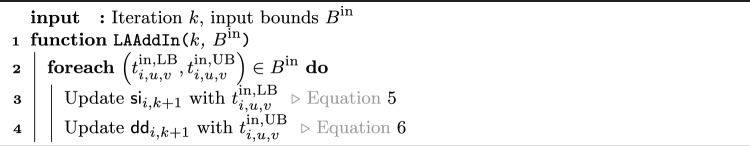
Updating problem $\tau _{i,k+1}$ with additional input constraints $B^{\textrm{in}}$

**Algorithm 4:**
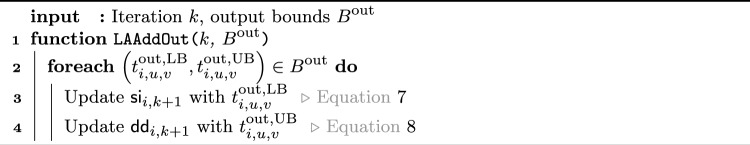
Updating problem $\tau _{i,k+1}$ with additional output constraints $B^{\textrm{out}}$

To update the problem tuple $\tau _{i,k+1}$ with the module-internal and received constraints, the LA uses Algorithm 3 for the input constraints and Algorithm 4 for the output constraints. Note that the problem tuple $\tau _{i,k+1}$ is initialised with the problem tuple $\tau _{i,k}$ of the previous iteration in function LAObtainBounds. Also module-internal bounds are already incorporated in LAObtainBounds.

##### Example 5.6

Recall Example 5.1. Figure 5b showed the propagation of the bound $t_{1,1,2,3}^{*\textrm{out},\textrm{LB}}$ with value 115 to module $n_{1}$. This bound is added as a sequence-independent setup time of 95 from $o_{2,4}$ to $o_{3,4}$ using Equation 7:

$$\begin{aligned} \mathsf{si}_{1,2}(o_{2,4}, o_{3,4}) = \max (\underbrace{115}_{t_{1,1,2,3}^{*\textrm{out},\textrm{LB}}} - \underbrace{20}_{\mathsf{prc}_{1}(o_{3,4})}, \underbrace{0}_{\mathsf{si}_{1,2}(o_{2,4}, o_{3,4})}) = 95 \end{aligned}$$

$$\blacksquare $$  

#### Schedule generation

The LA uses Algorithm 5 to generate the local schedule. The (partial) function ${\mathsf{s}}$, defined as ${\mathsf{s}}: J \hookrightarrow \mathbb {R}$, is a parameter, setting a lower bound on the start time of the first operation for each job for which it is defined.

**Algorithm 5:**
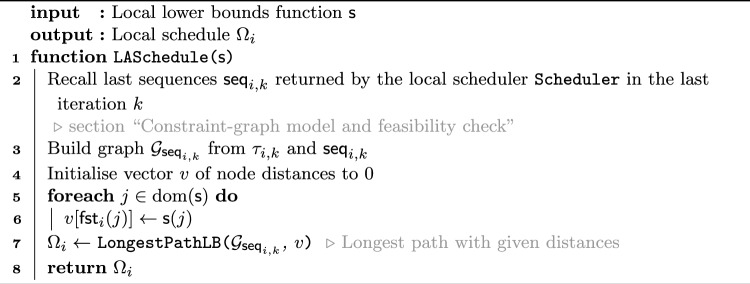
Generating local $\Omega _{i}$ with lower bounds ${\textsf{s}}$

The ASAP schedule is constructed using the longest path algorithm. The sequences that the local scheduler returns during the final iteration of the convergence step of Algorithm 1 form the graph $\mathcal {G}_{\mathsf{seq}_{i,k}}$ in lines 2 to 3. Then, Algorithm 5 sets the distances of the longest path to 0 for all nodes (line 4), excluding the distances for the nodes of the first operation of every job $j$ in the domain of $\mathsf{s}$ that are set to the value of ${\mathsf{s}}(j)$ (lines 5 to 6).

Finally, the LongestPathLB function takes the graph $\mathcal {G}_{\mathsf{seq}_{i,k}}$ and the lower bounds $v$ and determines, for each operation $o_{1}$ in $O_{i}$, the maximum over all $o_{2}$ in $O_{i}$ of $v[ o_{2}]$ plus the length of the longest path from $o_{2}$ to $o_{1}$. This provides the ASAP schedule of Definition 1 for the given machine sequences, as any operation starting time smaller than the computed longest paths will violate some constraints. The longest paths provide the smallest values that can satisfy the constraints. This computation is similar to the one used by van Pinxten et al. (2017).

##### Example 5.7

Recall Example 5.3. Figure 6b shows the schedule $\Omega _{2}$ of module $n_{2}$ as the values inside the nodes in the graph. This schedule is generated using Algorithm 5 and using as start-time lower bounds ${\mathsf{s}}(1) = 285$, ${\mathsf{s}}(2) = 320$, and ${\mathsf{s}}(3) = 435$. Inside Algorithm 5, the start-time lower bounds are used to initialise the longest-path algorithm that finds the longest distances from the source nodes ($o_{1,1}$, $o_{2,1}$, and $o_{3,1}$) to the other nodes in the graph $\mathcal {G}_{\mathsf{seq}_{2,6}}$ built from the problem $\tau _{2,6}$ and the sequences $\mathsf{seq}_{2,6}$ from Iteration 6 (the last iteration before schedule generation). $\blacksquare$

### Requirements on the LAs and local schedulers

Because the LAs and local schedulers are typically implemented by manufacturers of the modules, we state the requirements they need to adhere to so that the SA can properly coordinate the schedules of all modules.

The local scheduler must provide a schedule either as start times $\Omega$ or through the sequences of operations per machine in the module $\mathsf{seq}$. The sequences $\mathsf{seq}$ are needed by the LA in Algorithm 2 to produce the bounds that the LAs provides to the SA. If the local scheduler provides start times $\Omega$, the LA extracts the sequences $\mathsf{seq}$ from these start times. This ensures that the approach is compatible with many existing schedulers.

After the SA finds consensus between the modules in Algorithm 1, the decision on the final schedule of the module can be done either by the LA by accepting the schedule generated in Algorithm 5 or by the local scheduler. In the second case, the local scheduler must take into account the lower bound on start times ${\mathsf{s}}$ that the SA passes as a parameter to the LAs in lines 22 to 27 of Algorithm 1 and it must provide an ASAP schedule $\Omega$. Thus, to implement the LA, a manufacturer must always implement Algorithms 2 to 4 and, additionally, Algorithm 5 if the LA generates the final schedule. In this paper, we fully described the scenario where the local scheduler provides the sequences $\mathsf{seq}$ and the final schedule is decided by the LA.

Note that the SA generates a global schedule for the full production line in the final part of Algorithm 1. This is primarily done to enable the proof of soundness given in the next subsection, Sect.  “Proof of soundness”. The SA does not need to be aware of the full global schedule. The only information that the SA needs are the completion times of the last operations of each job processed by a module. This suffices to compute the lower bounds ${\mathsf{s}}$ in line 25 of Algorithm 1.

### Proof of soundness

We prove that if Algorithm 1 terminates at line 28, the production-line schedule $\Omega$ returned is feasible. After convergence (at line 22), all the modules are aware of their neighbours’ constraints. Consequently, it becomes possible to create mutually compatible schedules. A monolithic system can take all the local sequences $\mathsf{seq}_{i}$, build the global graph $\mathcal {G}_{\mathsf{seq}}^{global}$ (Sect. “Global constraint-graph model”), and obtain its ASAP schedule. However, since we work on a distributed system, we need to generate the schedules of the modules one by one. Therefore, we prove that the schedule obtained by following lines 22 to 27 of Algorithm 1 is the same as the one that is obtained by a monolithic system.    

To prove this, we first show that, after convergence, there are no positive cycles in the global graph $\mathcal {G}_{\mathsf{seq}}^{global}$ (Corollary 1). Then, we prove that the lengths of the longest paths are the same between the local graphs $\mathcal {G}_{\mathsf{seq}_{i}}$ and the global graph $\mathcal {G}_{\mathsf{seq}}^{global}$ for all pairs of nodes in each local graph $\mathcal {G}_{\mathsf{seq}_{i}}$ (Lemma 2). Finally, we prove that, with these properties, the algorithm generates the same ASAP schedule using the start times ${\mathsf{s}}$ and the local sequences $\mathsf{seq}_{i}$ as the global ASAP schedule of all the sequences $\mathsf{seq}_{i}$ (Lemma 3). Together, these results lead to the main result stated in Theorem 1, namely that the production-line schedule returned by Algorithm 1 is feasible.                

#### Lemma 1

If, after convergence of Algorithm 1, there is a cycle in the global graph $\mathcal {G}_{\mathsf{seq}}^{global}$ that spans more than one module, where $\mathsf{seq}$ refers to all the sequences after the last iteration of the convergence step of Algorithm 1, there exists also a cycle with a length at least as large as the original cycle that spans one module less.

#### Proof

Let there be such a cycle spanning more than one module. Let ($n_{i}, n_{i+1}, \ldots , n_{i+j}$) be the modules that the cycle spans. Then, all paths part of the cycle that go through the first module, $n_{i}$, have edges $( o_{1}, b, o_{2})$ in the second module $n_{i+1}$, whose weights are at least the lengths of the longest paths from $o_{1}$ to $o_{2}$ because of the propagation of bounds explained in Sects. “Constraint propagation” and “Incorporating constraints”. By replacing these paths with the corresponding edges in $n_{i+1}$, an equivalent cycle is obtained with a weight at least that of the original cycle but spanning one module less ($n_{i+1},\ldots ,n_{i+j}$). $\square$

It follows then from (repeatedly using) Lemma 1 that if a positive cycle exists in the global graph $\mathcal {G}_{\mathsf{seq}}^{global}$, then there exists also a positive cycle in the local graph $\mathcal {G}_{\mathsf{seq}_{i}}$ of some module $i$, implying that the local scheduling problem for module $i$ is infeasible. Thus, if a positive cycle exists in the global graph $\mathcal {G}_{\mathsf{seq}}^{global}$, Algorithm 1 terminates at line 9, without converging.    

#### Corollary 1

If Algorithm 1 converges at line 17, then the global graph $\mathcal {G}_{\mathsf{seq}}^{global}$ does not contain a positive cycle.    

A feasible schedule of a re-entrant flow-shop problem $\tau _{i}$ with sequence $\mathsf{seq}_{i}$ exists if and only if there are no positive cycles in the graph $\mathcal {G}_{\mathsf{seq}_{i}}$ built from the sequences $\mathsf{seq}_{i}$ and the tuple $\tau _{i}$. If a feasible schedule $\Omega _{i}$ exists, then the ASAP schedule exists and is unique. The ASAP schedule with the lower bound function ${\textsf{s}}$ used in lines 22 to 27 assigns a starting time $\Omega _{i}(o)$ to all operations $o$ in $O_{i}$ taking into account the lower bounds in ${\mathsf{s}}$. Since both the local and global ASAP schedules are obtained by finding the lengths of longest paths, we prove that these lengths are the same between the local graphs $\mathcal {G}_{\mathsf{seq}_{i}}$ and the global graph $\mathcal {G}_{\mathsf{seq}}^{global}$.    

#### Lemma 2

After convergence of Algorithm 1, the length of the longest path between two nodes in each local graph $\mathcal {G}_{\mathsf{seq}_{i}}$ is the same as the length of the longest path between two equivalent nodes (that is, the nodes representing the same operations of the same module) in the global graph $\mathcal {G}_{\mathsf{seq}}^{global}$.

#### Proof

We prove the lemma by contradiction. Assume that the length $l_{\mathsf{seq}_{i}}$ of the longest path from $o_{1}$ to $o_{2}$ in the local graph $\mathcal {G}_{\mathsf{seq}_{i}}$ is not equal to the length $l_{\mathsf{seq}}^{global}$ of the longest path between the same pair of operations in the global graph $\mathcal {G}_{\mathsf{seq}}^{global}$. If this is the case, there are two possible options:

$\boldsymbol{l_{\mathsf{seq}_{i}} > l_{\mathsf{seq}}^{global}}$. In this case, because the global graph $\mathcal {G}_{\mathsf{seq}}^{global}$ is built from all the local graphs $\mathcal {G}_{\mathsf{seq}_{i}}$ and the transfer constraints CT1 and CT2 (Sect. “Global constraint-graph model”), for each edge in $\mathcal {G}_{\mathsf{seq}_{i}}$ there exists an edge in $\mathcal {G}_{\mathsf{seq}}^{global}$ between the same pair of operations with the same weight. Thus, the longest path from $o_{1}$ to $o_{2}$ in the global graph $\mathcal {G}_{\mathsf{seq}}^{global}$ is at least as long as the longest path from $o_{1}$ to $o_{2}$ in the local graph $\mathcal {G}_{\textsf{seq}_{i}}$. This contradicts the fact that $l_{\mathsf{seq}_{i}} > l_{\mathsf{seq}}^{global}$.    

$$\boldsymbol{l_{\mathsf{seq}_{i}} < l_{\mathsf{seq}}^{global}}$$. If this is the case, there must exist a path with length $$l_{\mathsf{seq}}^{global}$$ that starts in $$o_{1}$$, leaves $$n_{i}$$ and enters it again, possibly multiple times, and finally ends in $$o_{2}$$. At least one of the detours leaving $$n_{i}$$ must be longer than the corresponding longest path inside the local graph $$\mathcal {G}_{\mathsf{seq}_{i}}$$. Without loss of generality, let operations $$ o_{1}'$$ and $$ o_{2}'$$ and job indices $$u$$ and $$v$$ be such that the detour from $$ o_{1}'$$ via $$\mathsf{fst}_{i}(j_{u})$$ through module $$n_{i-1}$$ via $$\mathsf{fst}_{i}(j_{v})$$ to $$ o_{2}'$$ is longer than the local longest path from $$ o_{1}'$$ to $$ o_{2}'$$. The length of the path from $$\mathsf{fst}_{i}(j_{u})$$ through module $$n_{i-1}$$ to $$\mathsf{fst}_{i}(j_{v})$$ is the translated bound $$t_{i,k,u,v}^{*\textrm{in},\textrm{LB}}$$, where $$k$$ is the last iteration of the main loop of Algorithm 1. Since we assume convergence of Algorithm 1, this translated bound must be equal to the length of the local longest path from $$\mathsf{fst}_{i}(j_{u})$$ to $$\mathsf{fst}_{i}(j_{v})$$, $$t_{i,k,u,v}^{\textrm{in},\textrm{LB}}$$. But from the above reasoning, it follows that $$t_{i,k,u,v}^{*\textrm{in},\textrm{LB}} > t_{i,k,u,v}^{\textrm{in},\textrm{LB}}$$, giving a contradiction. Considering the output side of the module instead of the input side, following a similar reasoning, we obtain that $$t_{i+1,k,u,v}^{*\textrm{out},\textrm{LB}} > t_{i+1,k,u,v}^{\textrm{out},\textrm{LB}}$$, also leading to a contradiction. So, we may conclude that Algorithm 1 has not converged if $$l_{\mathsf{seq}_{i}} < l_{\mathsf{seq}}^{global}$$.

Concluding, we reached a contradiction in both scenarios. Thus, proving the lemma. $\square$

We now define the ASAP schedule of the production-line problem $P$ and in Lemma 3 we prove that the schedule obtained after line 27 of Algorithm 1 is the ASAP schedule of the derived production-line problem $P_{\mathsf{MB}^{\textrm{in}},\mathsf{MB}^{\textrm{out}}}$ taking into account the bounds $\mathsf{MB}^{\textrm{in}}$ and $\mathsf{MB}^{\textrm{out}}$ computed in the last iteration $k$ of the main loop of Algorithm 1. $P_{\mathsf{MB}^{\textrm{in}},\mathsf{MB}^{\textrm{out}}}$ has the same modules and transfer points as the production line $P$, but each module $n_{i}\in N$ is equipped with flow-shop problem $\tau _{i,k}$ from the last iteration of Algorithm 1 instead of $\tau _{i}$.        

#### Definition 4

The ASAP schedule of a production-line problem $P$ and the sequences $\mathsf{seq}_{i}$ for all $n_{i}$ in $N$ is defined as a feasible schedule $\Omega$ where the start times of the operations of each machine $m$ in $M_{i}$ follow the order of the sequence $\mathsf{seq}_{i}(m)$ (i.e. $\Omega (\mathsf{seq}_{i}(m)(j_1)) < \Omega (\mathsf{seq}_{i}(m)(j_2))$ if and only if $j_1 < j_2$), and $\Omega (o)$ is minimal for all $o$ in $O$.

#### Lemma 3

The schedule $\Omega$ obtained after lines 22 to 27 of Algorithm 1 is the ASAP schedule for the production line $P_{\mathsf{MB}^{\textrm{in}},\mathsf{MB}^{\textrm{out}}}$ using the sequences $\mathsf{seq}_{i}$ and taking into account bounds $\mathsf{MB}^{\textrm{in}}$ and $\mathsf{MB}^{\textrm{out}}$ for all $n_{i}$ in $N$ of the last iteration of the main loop of Algorithm 1.

#### Proof

Because the process of lines 22 to 27 goes through an iterative process from the first module to the last, we prove it by induction. It is important to note that all of the modules have feasible schedules, because of Corollary 1. Furthermore, recall from Sects. “Constraint-graph model and feasibility check” and “Global constraint-graph model” that for every operation $o$ in the production line, the start time $\Omega (o)$ in the ASAP schedule $\Omega$ corresponds to the longest path in constraint graph $\mathcal {G}_{\mathsf{seq}}^{global}$ from the first operation of the first job in module $n_{1}$ to $o$.

 As a base case, consider the first module $n_{1}$ in the production line. Because the lower bounds defined by ${\mathsf{s}}$ are 0 for all first operations of all jobs performed by module $n_{1}$, and because of Lemma 2, the ASAP schedule for all module $n_{1}$-operations in the production line $P_{\mathsf{MB}^{\textrm{in}},\mathsf{MB}^{\textrm{out}}}$ is determined by the lengths of the longest paths from the first operation of the first job to all the others in the local $\mathcal {G}_{\mathsf{seq}_{i}}$. This is precisely what method call $LA_{1}$.LASchedule() in line 23 of Algorithm 1 provides. Hence, for all $n_{1}$-operations, $\Omega$, as computed in lines 22 to 27 of Algorithm 1, is the ASAP schedule.  

For the inductive step, considering module $n_{i}$ with $i>1$, assume that the start time of an operation $o$ of the previous module $n_{i-1}$ in de global ASAP schedule, $\Omega (o)$, equals the length of the longest path from the first operation of the first job in the first module to $o$ in the global graph $\mathcal {G}_{\mathsf{seq}}^{global}$. Observe that in line 26 of Algorithm 1 the parameter ${\mathsf{s}}$ passed to LASchedule contains, for the first operation $\mathsf{fst}_{i}(j)$ of each job $j$, the length of the longest path from the first operation in the system to that operation $\mathsf{fst}_{i}(j)$. For all the other operations $o$ in the module $n_{i}$, the longest path can be divided into (1) the path that goes from the first operation in the system to a first operation $\mathsf{fst}_{i}(j)$ in the module, for the first time, and (2) the path that goes from $\mathsf{fst}_{i}(j)$ to the operation $o$ of module $n_{i}$ whose longest path we are searching. The length of path (1) is, by induction, the value stored in the parameter ${\mathsf{s}}$. Thus, the length of the longest path from the first operation of the system to $o$ in the global graph $\mathcal {G}_{\mathsf{seq}}^{global}$ is, using Lemma 2 again, found by finding the largest value among all $j$ of the sum of ${\mathsf{s}}(\mathsf{fst}_{i}(j))$ and the length of all the longest path from $\mathsf{fst}_{i}(j)$ to $o$ in the local graph $\mathcal {G}_{\mathsf{seq}_{i}}$. This is precisely what the method LASchedule() in line 26 of Algorithm 1 does. Hence, also for module $n_{i}$, we may conclude that the start time $\Omega (o)$ in schedule $\Omega$ corresponds to the longest path in $\mathcal {G}_{\mathsf{seq}}^{global}$ from the first operation in the system to $o$. Thus, also for module $n_{i}$ we may conclude that $\Omega$, as computed in lines 22 to 27 of Algorithm 1, provides the ASAP schedule for production line $P_{\textsf{MB}^{\textrm{in}},\mathsf{MB}^{\textrm{out}}}$. $\square$            

#### Theorem 1

The schedule $\Omega$ obtained after lines 22 to 27 of Algorithm 1 is a feasible schedule for the production-line problem $P$.

#### Proof

The production-line problem $P_{\mathsf{MB}^{\textrm{in}},\mathsf{MB}^{\textrm{out}}}$ is derived from the original problem $P$ by adding constraints. Thus, any feasible solution of the derived problem is a feasible solution of the original problem. Thus, the desired result follows immediately from Lemma 3. $\square$

### Quantifying the performance of modular schedulers

In the following, we assess the performance of our distributed scheduling framework. In this subsection, we establish the metrics used for evaluation and comparison of modular scheduling solutions. Our focus is on three metrics: relative makespan, schedulability ratio, and execution time.

#### Definition 5

*((Relative) makespan)* Given a production line $P$ and a feasible schedule $\Omega$, the makespan of the schedule is the time between the start of the first operation in the system and the completion of the last operation in the system:

$$\begin{aligned} \mathsf{makespan}(P, \Omega ) = \max _{o \in O} (\Omega (o) + \mathsf{prc}(o)) - \min _{o \in O} \Omega (o) \end{aligned}$$

Given a base algorithm and a comparison algorithm, with makespans $\text {m}_B$ and $\text {m}_C$ respectively, the relative makespan between the base and the comparison is ${\text {m}_C}/{\text {m}_B}$.

#### Definition 6

*(Schedulability ratio)* Given a set of problems $\mathcal {P}$ to evaluate, the schedulability ratio is the ratio between the number of problems for which a feasible solution has been found and the total number of problems to solve.

$$\begin{aligned} \mathsf{schedulability\_ratio}(\mathcal {P}) = \frac{\left| \mathsf{solved}(\mathcal {P})\right| }{\left| \mathcal {P}\right| } \end{aligned}$$

#### Definition 7

*(Scheduler execution time)* The execution time of a scheduler on a given scheduling problem is the total amount of CPU time that the scheduler requires to execute. For a modular scheduler, this includes the CPU time that each local scheduler requires to solve all the local problems.

### Some concluding remarks

We designed a distributed scheduling method that propagates constraints between multiple schedulers in a production line. The structure is a hierarchical MAS architecture with the SA acting as a coordinator between LAs. We explained how the constraints are obtained, translated, propagated and incorporated in each module. We have shown that convergence of the distributed algorithm provides a globally feasible schedule for the full production line.

The quality of the global schedules generated depends both on the MAS design and on the quality of the local schedules. As long as it is possible to obtain bounds for subsequent jobs and sequences of operations, each module can use schedulers with different heuristics, such as for instance BHCS or MNEH discussed in Sect. “Related work”, and combine their strengths, allowing scheduling flexibility and design freedom in the design of production lines. We experimentally investigate schedule quality in Sect. “Experimental evaluation”. The next section first provides some background on two important design decisions in the proposed MAS, namely the bounds-propagation strategy and the decision to include a limit on the number of iterations of the main loop of Algorithm 1.

The presented approach can be applied to non-cyclic sequential module patterns with fixed-output order of jobs across modules. One could consider more general topologies, such as parallel modules. The SA would need to be aware of the destination of each job at every transfer point, and of the order of the jobs in the source and destination modules. This would allow the SA to send the bounds to the appropriate modules. Relaxing the fixed-output-order constraint (C6) would require updating the consensus mechanism. In such an updated mechanism, each module could propose a job order, and the SA could heuristically choose one that is globally near-optimal. Such extensions to other topologies and relaxing the ordering constraint are interesting topics for further work.

## Design decisions

### Bounds-propagation strategy

In Algorithm 1, the SA gathers and propagates bounds to other modules using a broadcast propagation strategy, where in each iteration of the propagation all constraints are communicated to all other modules. Moreover, the SA initially only propagates lower bounds; upper bounds are only propagated after initial convergence on the lower bounds. These choices impact the speed of convergence and the quality of the obtained solutions. It is important to find a good balance between convergence speed and solution quality. Related to this is the choice for the iteration limit $k^{\max {}}$. The following subsections discuss the propagation of lower and upper bounds, the propagation strategy itself, and the setting of $k^{\max {}}$ in some more detail.

#### Lower and upper bounds propagation

As previously explained, the convergence of Algorithm 1 follows a two-step process: (1) propagate lower bounds only and (2) propagate both lower and upper bounds. Propagating both lower and upper bounds immediately reduces the space of feasible solutions for the involved LA too fast, as every LA is limited to the space defined by those bounds. Thus, we defer the propagation of upper bounds until lower-bound convergence has been achieved. One may in fact wonder whether it is needed to propagate upper bounds at all? We are interested in ASAP schedules, so it is interesting to consider the option to only propagate lower bounds. But it turns out that propagation of upper bounds is required as without convergence on upper bounds as well, it is possible for Algorithm 1 to produce an infeasible schedule. This is shown in Example 6.1.

##### Example 6.1

In this example, we show that without propagating upper bounds, it is possible for Algorithm 1 to terminate but produce local schedules that violate the transfer constraints.

We define a production line with three modules $$n_{1}$$, $$n_{2}$$, and $$n_{3}$$, and machines $$M_{1} = \{m_{1}\}$$, $$M_{2} = \{m_{2}, m_{3}\}$$, and $$M_{3} = \{m_{4}\}$$. We schedule three jobs $$ J = \{j_{1}, j_{2}, j_{3}\}$$. Each job $$j_{u}$$ has the following operations in each module: $$\mathsf{ops}_{1}(j_{u}) = \{o_{u,1}\}$$, $$\mathsf{ops}_{2}(j_{u}) = \{o_{u, 1}, o_{u, 2}\}$$, and $$\mathsf{ops}_{3}(j_{u}) = \{o_{u, 1}\}$$. The mapping of operations for each job $$j_{u}$$ is $$\mathsf{mch}_{1}( o_{u,1})$$$${}= m_{1}$$, $$\mathsf{mch}_{2}( o_{u,1})$$$${}= m_{2}$$, $$\mathsf{mch}_{2}( o_{u,2})$$$${}= m_{3}$$, and $$\mathsf{mch}_{3}( o_{u,1})$$$${}= m_{4}$$. The processing time of all operations is $$1$$, i.e., for all $$o$$ in $$O_{1}$$, $$O_{2}$$ and $$O_{3}$$, $$\mathsf{prc}(o) = 1$$. The sequence-dependent setup time for all machines is $$1$$, i.e., for every pair of operations $$o_{1}$$ and $$o_{2}$$ such that $$\mathsf{mch}_{i}( o_{1}) = \mathsf{mch}_{i}( o_{2})$$, we have $$\mathsf{sd}_{i}(o_{1}, o_{2}) = 1$$.

**Fig. 8:**
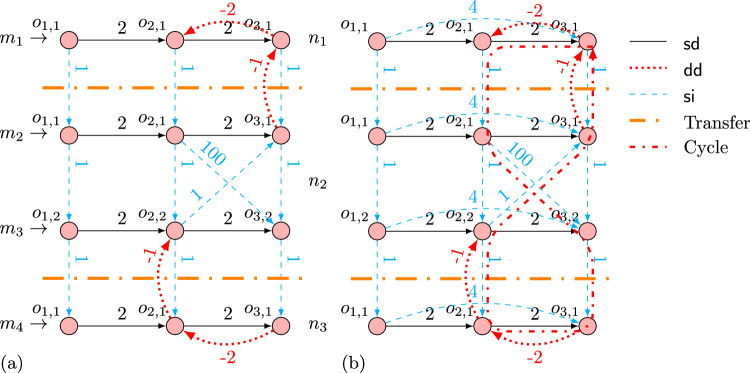
Constraint graphs for Example 6.1.** a** shows a constraint graph of the input problem with trivial sequences and** b** shows the graph after convergence. The red dashed and dotted line in **b** show the positive cycle that exists after termination and the horizontal edges from job $j_{1}$ to job $j_{3}$ are the added bounds

We add the sequence-independent setup times $\mathsf{si}_{2}(o_{2,1}, o_{3,2}) = 99$ and $\mathsf{si}_{2}(o_{2,2}, o_{3,1}) = 0$, and the due dates $\mathsf{dd}_{1}(o_{2,1}, o_{3,1}) = \mathsf{dd}_{3}(o_{2,1}, o_{3,1}) = 1$. For the transfer points $T_{1}$ and $T_{2}$, we set the transfer setup time to $\mathsf{ts}_{1}(j_{u}) = \mathsf{ts}_{2}(j_{u}) = 0$ for all $j_{u}$ in $J$ and the transfer due dates $\mathsf{td}_{1}(j_{3}) = \mathsf{td}_{2}(j_{2}) = 0$. A constraint-graph representation of these constraints can be seen in Fig. 8a, assuming machine sequences for all machines that process all operations of all jobs in order of the job index. These are in fact the (trivial) initial machine sequences that the LAs come up with when executing Algorithm 1, using BHCS (van Pinxten et al., 2017) as a local scheduler.

When scheduling the full production line of this example with Algorithm 1*without propagating upper bounds*, the algorithm terminates after two iterations of the main loop. The machine sequences do not change in the process. The only extra constraints added after termination are sequence-independent setup times $\mathsf{si}_{i}(o_{1,i}, o_{3,i}) = 3$ from each operation of $j_{1}$ to the same operation of $j_{3}$, shown in Fig. 8b.    

However, a positive cycle exists in the global graph of Fig. 8b, as indicated by the red dashed and dotted line. Given that the graph has a positive cycle, no feasible global schedule for the full production line exists. The positive cycle includes the sequence-independent setup constraint between operations $o_{2,1}$ and $o_{3,2}$. This constraint requires a big gap between the start of processing job $j_{2}$ and completing job $j_{3}$ in module $n_{2}$. But such a gap is not possible in the other two modules, because of the due-date constraints in those modules in combination with the transfer due dates. This large setup constraint between $o_{2,1}$ and $o_{3,2}$ is never propagated from module $n_{2}$ to the other modules, because it is not part of any path between any pair of input operations or any pair of output operations of $n_{2}$.

Returning to Algorithm 1 (without upper bound propagation), this means that generating the full schedule in lines 22 to 27 fails. The last module is not able to generate a schedule, because, given the transfer due date for operation $o_{2,1}$, it is forced to separate operations $o_{2,1}$ and $o_{3,1}$ further than allowed by its local due date.

This issue can be resolved by propagating upper bounds. When running Algorithm 1 as proposed (so, including upper bound propagation), two extra edges $( o_{3,1}, -2, o_{2,1})$, and $( o_{3,2}, -2, o_{2,2})$ are included in the local constraint graph of module $n_{2}$. This creates a positive cycle inside module $n_{2}$. As a result, Algorithm 1 terminates at line 9 without returning a schedule, in line with the earlier observation that no feasible schedule exists. $\blacksquare$

#### Propagation strategy

**Fig. 9:**
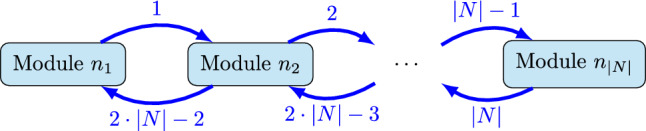
Cocktail-shaker propagation strategy

As explained, Algorithm 1 propagates bounds to other modules using a broadcast propagation strategy. A potential risk of this broadcast strategy is that the bounds of modules further apart in the production line might not be compatible with each other. It is interesting to compare the broadcast strategy with a strategy that strives for global compatibility of constraints during constraint propagation in each iteration. We designed such an alternative strategy, dubbed the *cocktail-shaker* propagation strategy, depicted in Fig. 9. Here, the SA collects the bounds of module $n_{1}$ and propagates them to module $n_{2}$. Module $n_{2}$ updates its local scheduling problem with the received bounds and produces its own bounds, that the SA then propagates to module $n_{3}$, and so on. This process continues until the last module. Then, the process is reversed, starting propagation from the last module, propagating constraints one module at a time till the first module is reached again. The complete propagation process from the first to the last module and back constitutes one iteration in Algorithm 1. This cocktail-shaker propagation, replacing line 7 to line 14 in Algorithm 1, repeats until consensus is reached, i.e., as before, until the bounds are the same as the bounds of the previous iteration. We compare the broadcast and cocktail-shaker strategies in our experimental evaluation.

#### Setting the iteration limit $k^{\max {}}$

The purpose of the $k^{\max {}}$ limit is to stop the scheduling when non-convergence is suspected. A value too small may prematurely stop Algorithm 1, reducing the overall quality. A lower bound on the minimum number of expected iterations (for the broadcast strategy) is $4(|N| - 1)$ as it requires $2(|N| - 1)$ iterations for information to propagate from the first to the last module and back, and there are two convergence processes. For the cocktail-shaker propagation strategy, the lower bound for $k^{\max {}}$ is 2, since one iteration already propagates constraints back and forth through the full production line. The actual value must be above the minimum number of expected iterations but it must also fit within the time budget of the application. Given that the time per iteration is relatively stable (from our experimental evaluation), a good estimate can be made from experiments with application-specific problem instances, observing the needed number of iterations. The value can also be adjusted online if all (or most) instances converge within an observed number of iterations.

### Termination analysis

Given that Algorithm 1 propagates both lower and upper bounds between modules, it may seem that eventually the algorithm must converge, either because a schedule is found or because the bounds cross each other. In the latter case, the algorithm must terminate because a lower bound that is higher than an upper bound creates an infeasible problem. However, without the upper limit $k^{\max {}}$ on the main loop of Algorithm 1, it is possible to craft a counterexample that does not terminate by alternating between different sequences in the main loop of the algorithm. Example 6.2 below shows this counterexample. Note that, since the number of possible scheduling sequences per module is finite, such a non-terminating scenario could be mitigated by disallowing the reuse of already considered and discarded sequences in each of the LAs. However, that would require considerably more analysis effort to be performed in the LAs. We therefore opted not to do this.

#### Example 6.2

In this example, we show how it is possible that Algorithm 1 does not terminate if there is no hard limit on the number of iterations.

**Fig. 10:**
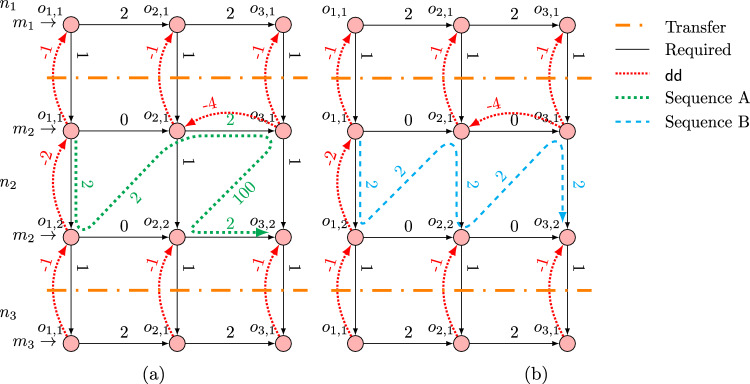
Constraint graph of Example 6.2 with two possible machine sequences. Sequence A is shown in **a** and sequence B in **b**

We define a production line with three modules $$n_{1}$$, $$n_{2}$$, and $$n_{3}$$ and machines $$M_{1} = \{m_{1}\}$$, $$M_{2} = \{m_{2}\}$$, and $$M_{3} = \{m_{3}\}$$. We schedule three jobs $$ J = \{j_{1}, j_{2}, j_{3}\}$$. Each job $$j_{u}$$ has the following operations in each module: $$\mathsf{ops}_{1}(j_{u}) = \{o_{u,1}\}$$, $$\mathsf{ops}_{2}(j_{u}) $$$${} = \{o_{u, 1}, o_{u, 2}\}$$, and $$\mathsf{ops}_{3}(j_{u}) $$$${}= \{o_{u, 1}\}$$. The mapping of operations for each job $$j_{u}$$ is the trivial one, $$\mathsf{mch}_{1}( o_{u,1}) = m_{1}$$, $$\mathsf{mch}_{2}( o_{u,j}) = m_{2}$$, and $$\mathsf{mch}_{3}( o_{u,1}) = m_{3}$$. The processing time of all operations is $$1$$. The sequence-dependent setup times for all machines are $$\mathsf{sd}_{i}(o_{1}, o_{2}) = 1$$ for all $$o_{1}$$ and $$o_{2}$$ with $$\mathsf{mch}_{i}( o_{1}) = \mathsf{mch}_{i}( o_{2})$$ except $$\mathsf{sd}_{2}(o_{3,1}, o_{2,2}) = 99$$. We add the due dates $$\mathsf{dd}_{2}(o_{1,1}, o_{1,2}) = 1$$, and $$\mathsf{dd}_{2}(o_{2,1}, o_{3,1}) = 3$$. For the transfer points $$T_{1}$$ and $$T_{2}$$, we set all transfer setup times and due dates to $$0$$, i.e., $$\mathsf{ts}_{1}(j_{u}) = \mathsf{ts}_{2}(j_{u}) = 0$$ and $$\mathsf{td}_{1}(j_{u}) = \mathsf{td}_{2}(j_{u}) = 0$$ for all $$j_{u}$$ in $$J$$. Figure 10 shows a constraint-graph representation of the production line, assuming the trivial machine sequences for modules $$n_{1}$$ and $$n_{3}$$, and two possible sequences for module $$n_{2}$$.        

In this setup, module $n_{2}$ keeps alternating between the two sequences and thus the bounds exchanged between modules keep changing. The values of these bounds are shown in Table 4. Assume, towards a counterexample, that, in the first iteration, module $n_{2}$ chooses scheduling sequence A, shown in Fig. 10a. Note that sequence A is not optimal. Sequence B shown in Fig. 10b is the optimal sequence. So, a normal scheduler would likely not select sequence A. We chose this sequence for illustration purposes, to obtain a small counterexample. Similar behaviours can be achieved with larger examples and schedulers looking for optimality.

**Table 4:** Values of the bounds of each module, as difference between start times for input bounds and completion times for output bounds, at the end of every iteration of Algorithm 1

Iteration ($k$)		1	2	3	4	5	6	7
$n_{i}$	Sequence	A	B	A	B	A	B	A
$n_{1}$	$t_{1,k,1,2}^{\textrm{out},\textrm{LB}}$	2	**4**	**104**	104	**206**	206	**308**
	$t_{1,k,1,3}^{\textrm{out},\textrm{LB}}$	4	**6**	**108**	108	**210**	210	**312**
	$t_{1,k,2,3}^{\textrm{out},\textrm{LB}}$	2	2	**4**	4	4	4	4
$n_{2}$	$t_{2,k,1,2}^{\textrm{in},\textrm{LB}}$	4	**104**	104	**206**	206	**308**	308
	$t_{2,k,1,3}^{\textrm{in},\textrm{LB}}$	6	**108**	108	**210**	210	**312**	312
	$t_{2,k,2,3}^{\textrm{in},\textrm{LB}}$	2	**4**	4	4	4	4	4
	$t_{2,k,1,2}^{\textrm{out},\textrm{LB}}$	104	104	**206**	206	**308**	308	**410**
	$t_{2,k,1,3}^{\textrm{out},\textrm{LB}}$	106	**108**	**210**	210	**312**	312	**414**
	$t_{2,k,2,3}^{\textrm{out},\textrm{LB}}$	2	**4**	4	4	4	4	4
$n_{3}$	$t_{3,k,1,2}^{\textrm{in},\textrm{LB}}$	2	**104**	104	**206**	206	**308**	308
	$t_{3,k,1,3}^{\textrm{in},\textrm{LB}}$	4	**106**	**108**	**210**	210	**312**	312
	$t_{3,k,2,3}^{\textrm{in},\textrm{LB}}$	2	2	**4**	4	4	4	4

The table heading shows the iteration count and the sequence used. Changes between iterations are highlighted in bold face

The purpose of the due dates $\mathsf{dd}_{2}(o_{1,1}, o_{1,2}) = 1$ and $\mathsf{dd}_{2}(o_{2,1}, o_{3,1}) = 3$ is to create a mutual dependency between $t_{2,k,1,2}^{\textrm{in},\textrm{LB}}$ and $t_{2,k,1,2}^{\textrm{out},\textrm{LB}}$ when using sequences A and B. In iterations with sequence A, the bound $t_{2,k,1,2}^{\textrm{in},\textrm{LB}}$ gets “amplified” by the setup time $\mathsf{si}_{2}(o_{3,1}, o_{1,2})=99$ and $t_{2,k+1,1,2}^{\textrm{out},\textrm{LB}} \approx t_{2,k,1,2}^{\textrm{in},\textrm{LB}} + 100$. After the first two iterations, the longest path from $o_{1,2}$ to $o_{2,2}$ with sequence A is the sequence of nodes $\langle o_{1,2}, o_{1,1}, o_{2,1}, o_{3,1}, o_{2,2}\rangle$ (because the weight $w$ of the edge $( o_{1,1}, w, o_{2,1})$ increases as a consequence of adding the $t_{2,k,1,2}^{\textrm{in},\textrm{LB}}$ bound). In iterations with sequence B, the value of the bound $t_{2,k,1,2}^{\textrm{out},\textrm{LB}}$ is propagated back to $t_{2,k+1,1,2}^{\textrm{in},\textrm{LB}}$ because the longest path from $o_{1,1}$ to $o_{2,1}$ with sequence B after the first two iterations involves the nodes $\langle o_{1,1}, o_{1,2}, o_{2,2}, o_{3,1}, o_{2,1}\rangle$ (because the weight $w$ of the edge $( o_{1,2}, w, o_{2,2})$ increases as a consequence of adding $t_{2,k,1,2}^{\textrm{out},\textrm{LB}}$). This process continues indefinitely.    

Table 4 shows the bound values during the first seven iterations. *Iteration 1*: the scheduler of module $n_{2}$ selects sequence A. The bound $t_{2,1,1,2}^{\textrm{out},\textrm{LB}}$ from $o_{1,2}$ to $o_{2,2}$ in $n_{2}$ is $104$ (from completion time of $o_{1,2}$ to completion time of $o_{2,2}$) and it is added as the setup time $\mathsf{si}_{2,2}(o_{1,2}, o_{2,2}) = 103$ (subtracting the processing time $\mathsf{prc}_{2}(o_{1,1}) = 1$). *Iteration 2*: the scheduler of module $n_{2}$ chooses sequence B. With this sequence and the added setup time $\mathsf{si}_{2,2}(o_{1,2}, o_{2,2}) = 103$ the input bound $t_{2,2,1,2}^{\textrm{in},\textrm{LB}}$ has the value 104 and gets added as the setup time $\mathsf{si}_{2,3}(o_{1,1}, o_{2,1}) = 103$. *Iteration 3*: the scheduler of module $n_{3}$ chooses sequence A. Now the output bound $t_{2,3,1,2}^{\textrm{out},\textrm{LB}}$ has the value 206. At this point, the output bound has already been amplified once so the following iterations just repeat this process without termination. $\blacksquare$

## Experimental evaluation

This section describes the experimental evaluation of our MAS approach. We integrate two state-of-the-art schedulers for re-entrant flow shops (BHCS (van Pinxten et al., 2017) and MNEH (Jeong & Kim, 2014)) into the MAS. The evaluation focuses on scalability and schedule quality, using the relative makespan, schedulability ratio, and scheduler runtime metrics defined in Sect. “Quantifying the performance of modular schedulers”, as well as reusability of local schedulers. We use a monolithic, exact scheduler to quantify the quality of our modular solution. The monolithic scheduler has complete knowledge, mimicking an ideal scenario, but cannot be applied for modular setups. We implemented the exact monolithic scheduler (MON) using constraint programming (CP). Typically, CP provides the most competitive solutions for this type of scheduling problems (Laborie et al., 2018). To implement the CP solution, we used IBM ILOG CP Optimizer 22.1.1.0 (IBM Corporation, 2023).

### Experiment design

We designed synthetic experiments for both homogeneous and heterogeneous production lines, drawing inspiration from an industrial case. Each type of production line includes eight different scenarios. Homogeneous lines consist of multiple instances of the same module, while heterogeneous lines feature modules with varying timings. Across scenarios, jobs vary in processing times, setup times, and due dates. For each type and scenario, we generate flow-shop problems with 5, 10, 20, 30, 40, 60, 80, 100, 150, 200, 250, and 300 jobs, and 2 to 10 modules in a production line, giving a total of 8640 problem instances. The processing times, setup times, and due-date values are based on the experiments by van Pinxten et al. (2017).

We implemented both the broadcast strategy presented in Algorithm 1 and the cocktail-shaker propagation strategy discussed in Sect. “Propagation strategy” as a C++ program. In this program, we also implemented both the BHCS and MNEH local schedulers of van Pinxten et al. (2017) and Jeong and Kim (2014), respectively. Additionally, as an evaluation of a fallback mechanism, we also implemented a third local scheduler, referred to as the “simple” scheduler, that generates naive schedules that are always feasible in our specific problem case. The simple scheduler schedules all the re-entrant operations of a job before scheduling the re-entrant operations of the next job. Thus, it never interleaves operations of multiple jobs. This is always possible because we do not have due dates between jobs; we only have due dates within jobs. For the sake of comparison between variants of our MAS approach, we implemented the bound $k^{\max {}}$ in Algorithm 1 as a 10-minute CPU time limit.

As mentioned, our evaluation focuses on schedule quality and scalability. Scalability is investigated in terms of the number of modules in a production line and the number of jobs to be scheduled. As quality metric, we consider the schedule makespan (Definition 5). We use the monolithic scheduler MON, which has complete knowledge of the production-line setup and jobs to be scheduled, as a reference. Ideally, MON can provide optimal schedules, given enough time. We also evaluate the schedulability ratio (Definition 6) of the various setups. All problem instances are feasible by construction. So, the schedulability ratio provides insight in how often a backup scenario (i.e. falling back to the naive schedule generated with the simple local schedulers) is needed because the MAS cannot find a solution.

To obtain a broad overview of the behaviour of our MAS, we ran both our MAS and MON with an execution time limit of 10 min of CPU time per instance, i.e., a bound on the scheduler execution time as defined in Definition 7. Considering these results, we then selected 1080 instances from the heterogeneous production lines and we ran them with a time limit of 60 min of CPU time per instance. We used the increased time to find more cases of optimal solutions with the exact scheduler MON and to test whether an increase of $k^{\max {}}$ in our MAS approach would allow more instances to reach consensus with the MAS. To compare the effect of different local schedulers, we also evaluated these 1080 instances with BHCS, MNEH, and combinations of these two schedulers as local schedulers and a 60 min CPU time limit per instance.

### Experiments results

This section first discusses the aggregated results of the 10-minute experiments to obtain a high-level overview of the behaviour of our MAS approach. Section “Scalability” then investigates the scalability of our approach in terms of the number of modules and the number of jobs. We discuss the 60-minute experiments in Sect. “Quality analysis” to understand the effect of having a larger time budget for scheduling challenging cases. Finally, we compare different local schedulers in Sect. “Reusing local schedulers”.

#### Overview

**Fig. 11:**
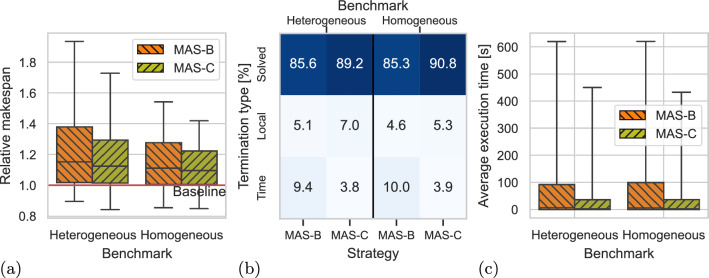
Evaluation results.** a** Relative makespan (lower is better) by benchmark type of our MAS with cocktail (MAS-C) and broadcast (MAS-B) propagation strategies, compared to MON.** b** Percentage of instances that were solved (Solved), ran into a local infeasibility (Local), or ran out of time (Time) by benchmark type and algorithm.** c** Average algorithm execution time by benchmark type. For both box plots, the whiskers represent the 5^th^ and 95^th^ percentile of all data

Figure 11 presents the aggregated results of all the 10-minute experiments. MAS-B and MAS-C refer to our MAS with BHCS as the local scheduler using the broadcast and cocktail constraint propagation strategies, respectively. We used BHCS in this first set of experiments because of its good trade-off between efficiency and quality, enabling the testing of many scenarios.

First, we compare the relative makespan (see Definition 5) of MAS-B and MAS-C using MON as a baseline in Fig. 11a. MON is expected to give near-optimal results. (Optimality is not guaranteed given the imposed run-time limit.) The relative makespan gives an indication of how good the solutions of the MAS are. The average relative makespan of MAS-C is 1.18 for heterogeneous production lines, 1.12 for homogeneous ones, and 1.15 overall. With MAS-B, it is 1.24, 1.15, and 1.20, respectively. These results are significantly better than the relative makespan using the simple local scheduler, which is 4.15 on average (irrespective of the constraint-propagation strategy). This confirms the importance of the local scheduler in the quality of the global schedules. The schedule quality is determined by the combination of the MAS constraint-propagation strategy and the used local scheduler.

As mentioned, it is also interesting to consider the schedulability ratio (Definition 6). We compare the two propagation strategies of our MAS with the BHCS local scheduler across the two benchmarks in Fig. 11b. There is no significant difference between the benchmarks. Instances that could not be scheduled by MAS-C became locally infeasible (6.1%), or reached the time limit $k^{\max {}}$ (3.9%). Similarly, for MAS-B, 4.8% of the instances became locally infeasible and 9.7% reached the time limit. Figure 11b shows the breakdown over the two benchmark types. The differences in local infeasibility and time-outs between both propagation strategies are interesting. MAS-C explicitly tries to propagate globally compatible constraints by going forward and backward through the modules in each iteration; MAS-B propagates constraints more aggressively without global alignment. The latter may limit scheduling freedom too much, in some cases leading to local infeasibility but often also leading to continued iterations until a time-out occurs. With the simple local backup scheduler, both MAS-C and MAS-B generated, as expected, a feasible schedule for all instances.

Finally, we evaluate the average execution time of MAS-B and MAS-C. Figure 11c shows a box plot of the execution times. In general, the execution time of MAS-C is lower than the execution time of MAS-B. This is due to the smarter propagation strategy, leading to local schedulers creating more compatible schedules in the first iterations. While the average and the median execution times are quite low, there are some instances that take a significant amount of time. As said previously, 3.9% of the instances for MAS-C and 9.7% for MAS-B reach the $k^{\max {}}$ limit. The size of the input problem has a strong impact on the execution time. This is further investigated in the next subsection.

#### Scalability

**Fig. 12:**
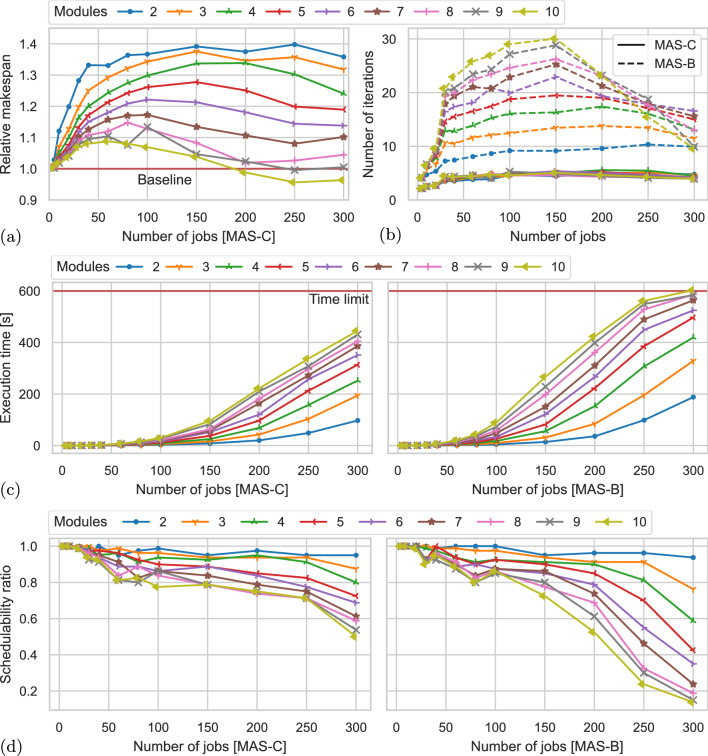
Scalability results while varying the numbers of jobs and modules.** a** Relative makespan (lower is better) of MAS-C.** b** Number of iterations comparing MAS-C and MAS-B.** c** Execution time in seconds of both MAS-C (left) and MAS-B (right).** d** Schedulability ratio of MAS-C (left) and MAS-B (right)

Figure 12 illustrates the behaviour of our MAS across different numbers of jobs and modules. In terms of relative makespan (Fig. 12a), the difference of MAS-C (which outperforms MAS-B) against the near-optimal monolithic solution becomes less significant as the number of modules increases. Increasing the number of jobs also seems to lower the gap, but less significantly. This can be related to the fact that MON is unable to find near-optimal solutions in the allocated time. This is further investigated in Sect. “Quality analysis” with the increased time budget of 60 min.

Regarding the number of iterations required by Algorithm 1 to converge, Fig. 12b, shows the iterations required by MAS-C and MAS-B. The difference in number of iterations between the two propagation strategies shows the effectiveness of MAS-C by only scheduling a module after it has received the constraints from its neighbour. In contrast, MAS-B requires a lot of iterations before the constraints of each module have reached all the other ones. However, note that a MAS-C-iteration covers a full forward and backward traversal of all modules, whereas a MAS-B-iteration exchanges bounds between all neighbouring modules only once. Thus, one iteration of MAS-B requires local scheduling $|N|$ times while one iteration of MAS-C requires local scheduling $2|N|$ times. For instances with more than 150 jobs and more than 5 modules, the number of iterations required by MAS-B goes down. This is caused by MAS-B reaching the maximum execution time of 10 min (see Fig. 12c). Thus, because the time per iteration is quite stable, only the instances that can be solved with a smaller number of iterations are solved within the execution time limit. Increasing the number of jobs or modules essentially has a linear effect on the execution time if solutions can be found within the time budget.

Figure 12d shows the schedulability ratio while varying the numbers of jobs and modules. In general, as the number of modules increases, the schedulability ratio goes down. This can be caused by incompatibilities arising from local scheduling decisions as more modules are involved in a production line. The same applies for an increased number of jobs. For MAS-B, part of the effect can be explained by reaching the $k^{\max {}}$ limit for the larger instances. Overall, MAS-C outperforms MAS-B because its propagation strategy tries to ensure global compatibility of the exchanged bounds.

#### Quality analysis

**Fig. 13:**
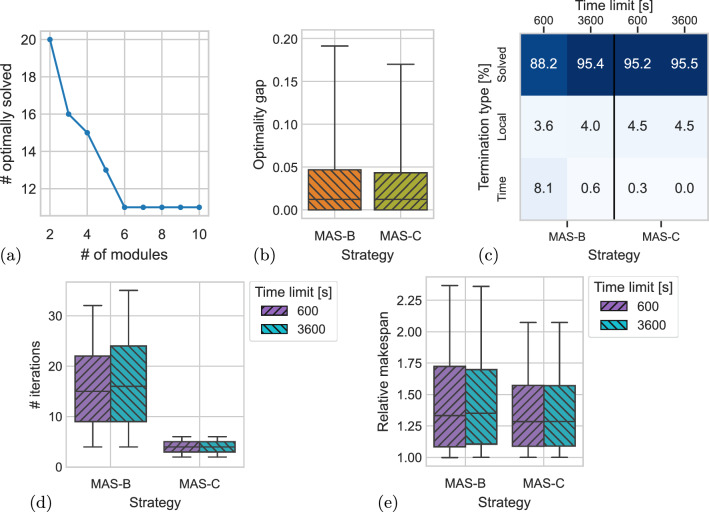
**a** Number of optimal solutions found with MON while varying the number of modules.** b** Box plot of the optimality gap (lower is better) between MAS-B, MAS-C, and the instance’s optimal solution for solutions known to be optimal.** c** Percentage of instances that were solved (Solved), ran into local infeasibility (Local), or ran out of time (Time) by propagation strategy and time limit.** d** Number of iterations required to converge for both MAS-B and MAS-C by time limit.** e** Relative makespan of MAS-B and MAS-C by time limit when compared to the solutions of MON using a time limit of 60 min

Next, we evaluate the differences between known optimal solutions and the solutions provided by our MAS, as well as the effects of a larger $k^{\max {}}$ limit. We evaluated a subset of 1080 instances of the heterogeneous benchmark with our MAS and time limits of 10 min and 60 min, using optimal solutions found by MON with a time limit of 60 min as reference.

We were able to find 119 optimal instances with MON, all for 10 jobs or less. Their distribution over the number of modules is shown in Fig. 13a. Both MAS-C and MAS-B were able to solve the same 42 out of the 119 instances (35%) to optimality. They solved the exact same instances due to the small problem size, since the differences between propagation strategies are not that visible with problems with 10 or less jobs. We also evaluate the optimality gap, which we define as $\frac{\text {makespan} - \text {optimal~makespan}}{\text {makespan}}$, in Fig. 13b. There is a small difference between MAS-C and MAS-B but both are relatively close to the optimum (0 gap) in most cases.

We also evaluated the schedulability for the 1080 instances when increasing the $k^{\max {}}$ limit. Figure 13c shows the termination types of each strategy with different time limits (10 and 60 min). When increasing the time limit from 10 min to 60 min, the schedulability ratio for MAS-B increases from 88.2% to 95.4% with only 0.6% reaching the time limit. This increase is caused by MAS-B successfully converging in more instances. Similarly, for MAS-C, it increased slightly from 95.2% to 95.5% without any instances still reaching the time limit. The rest of the instances that could not be scheduled were because the problems became locally infeasible. This effect was expected from the earlier analysis of Fig. 12a and c. It can now be observed in Fig. 12d that the 95-th percentile of the number of iterations is higher with a 60-minutes time budget. A 10-minutes time limit may lead to unschedulability by a false positive of non-convergence.

Finally, we compare in Fig. 13e the changes in relative makespan when increasing the iterations limit. For MAS-C, there is no significant difference as almost all of the instances were already solved within the time limit of 10 min. For MAS-B, there is a slight visible change caused by more instances being solved. Thus, we can conclude that the time limit of 10 min is mostly sufficient for MAS-C but too low for MAS-B.

#### Reusing local schedulers

Finally, as a way to test the applicability of our method to different local schedulers, we implemented the MNEH scheduling algorithm and used it in our MAS. We created two different scenarios. In one scenario, all the modules in the production line use MNEH as the local scheduler (MAS-MNEH). In the other scenario, we used both BHCS and MNEH in an interleaved manner (MAS-INTL). That is, if one module uses BHCS as the local scheduler, then the following one uses MNEH and vice versa. These are the most adverse conditions for our MAS for testing multiple local schedulers in a single production line. We then combined the results with the scenario from the previous section using only BHCS (MAS-BHCS). All setups use the cocktail constraint propagation, because the earlier experiments show that this strategy outperforms the broadcast strategy. We used the problem instances of Sect. “Quality analysis” for these experiments.

**Fig. 14:**
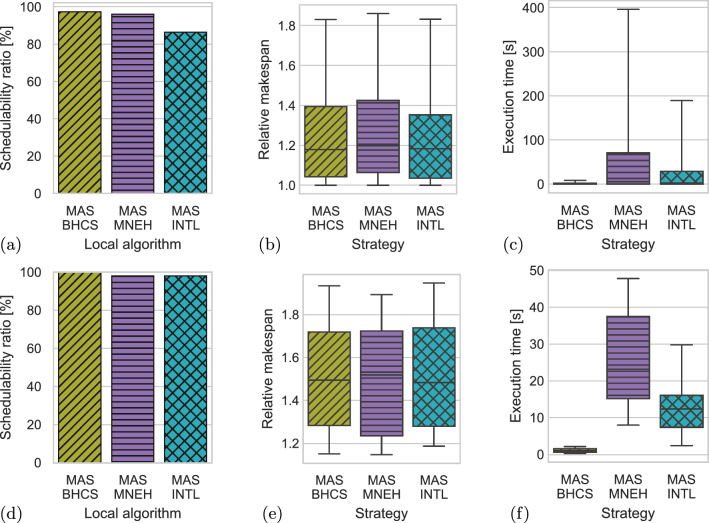
**a**,** b**, and** c**: results with instances up to 100 jobs and 10 modules.** d**,** e**, and** f**: results with instances up to 60 jobs and 6 modules. **a** and **d**: schedulability ratio of each scheduler. **b** and **e**: relative makespan of each scheduler when compared to MON. **c** and **f**: execution time box plot of each scheduler. For the box plots, the whiskers represent the 5^th^ and 95^th^ percentile of all data

Figure 14 shows the results of our experiments with these local schedulers. The top row shows results for instances up to and including 100 jobs and 10 modules; the bottom row shows results for instances up to and including 60 jobs and 6 modules. MNEH is a slower scheduler than BHCS. Since the goal is to illustrate the reuse of local schedulers and to compare the results for different local schedulers, we focus the discussion on problem instances that can be handled by both local schedulers. The 100 jobs/10 modules instances are the biggest instances that can be evaluated without any the three schedulers (MAS-BHCS, MAS-MNEH, and MAS-INTL) running out of time on the 60-minute time limit (only one MAS-MNEH instance ran out of time). Up to 60 jobs and 6 modules, almost all problem instances can be solved with both local schedulers (without invoking the simple backup scheduler). These cases illustrate the best conditions for the local schedulers to work. In this way, we can make a proper comparison of these local schedulers in scenarios in which they perform the best.

In terms of schedulability, for up to 100 jobs (Fig. 14a), MAS-BHCS and MAS-MNEH perform very similarly while the combination of both schedulers in MAS-INTL performs a bit worse. This is caused by the schedules between two neighbouring modules, derived by different local schedulers, not being completely compatible, making it more difficult for the MAS to find consensus. Interestingly, this does not happen for smaller instances (Fig. 14d).

Regarding relative makespan when compared to the MON, all schedulers perform very similarly. Interestingly, MAS-MNEH seems to perform slightly worse than the other two schedulers for large instances (Fig. 14b) but not for the smaller ones (Fig. 14e). Also, the interleaving of MNEH and BHCS in MAS-INTL seems to provide solutions with quality closer to BHCS than to MNEH.

For execution time, MAS-BHCS requires much less execution time while MAS-MNEH requires significant execution time. This is to be expected since BHCS is an online scheduler while MNEH is not. Nevertheless, 98.7% of the instances of MAS-MNEH finished in less than 1 min for the up to 60 jobs and 6 modules benchmark. MAS-INTL is in between the other two since half of the modules use BHCS and the other half MNEH.

### Discussion

In the previous three subsections, we evaluated our MAS approach with various propagation strategies and various local schedulers for scheduling modular production lines, using results from a monolithic CP solution as reference. It is important to observe that the latter is not a modular solution and requires complete knowledge of the production line. This is often not feasible when production lines are built from systems of different vendors, as in chip manufacturing and production printing (Traganos et al., 2020; Yang et al., 2008). In today’s practice, such production lines are typically loosely coupled, with buffers between systems of different vendors and no schedule alignment among these systems. In such setups, our modular MAS approach, that allows the integration of different modules and schedulers, may provide an attractive solution. The results show that the MAS provides for heterogeneous setups schedules with on average 15% larger makespan than the near-optimal monolithic scheduler, which is likely to be an improvement, productivity-wise, compared to a loosely coupled production line with buffering.

Considering the runtime, the MAS approach (with the BHCS local scheduler) is competitive as it can schedule large instances of 10 modules and 300 jobs in less than 8 min on average (about 1.5 s per job). In a modularised production line, the search space of each module is smaller compared to the global search space of a monolithic setup. For schedulers that use Bellman-Ford, such as BHCS, a reduction in the size of the constraint-graph model significantly benefits speed. Furthermore, the MAS is able to leverage existing heuristics for local modules. The scalability of our solution is evident from Fig. 12.

When comparing the different constraint propagation strategies, the cocktail propagation strategy is faster, it has a higher schedulability ratio, and it produces schedules with smaller makespans than the broadcast strategy. However, it is important to note that the reported execution times refer to a single-core implementation. In practice, since modules typically have their own compute resources, the broadcast strategy is naturally parallelised, while cocktail cannot be parallelised, because each module evaluates its bounds (LAObtainBounds) separately in order. This would reduce the practical execution time of the broadcast strategy by approximately a factor equal to the number of modules, with a constant offset for the SA computations and for communication between the SA and the modules. This speed-up may be interesting for setups where the execution time of the scheduler is critical and it is a good compromise to have faster results at the expense of slightly worse makespan. Propagation strategies may be further customised to meet the needs of a particular system setup.

## Conclusions

The distributed multi-agent flow-shop scheduling framework presented in this paper enables the coordination of multiple production modules within a tightly coupled production line in which jobs are processed in a fixed, sequential order. It abstracts the behaviour of a module into bounds on start and completion times of jobs that are shared among the modules. We have proven that the approach is sound, for non-cyclic module patterns with fixed output order, in the sense that the scheduled handovers of work in progress between modules are compatible with the local module schedules. The strength of our MAS lies in the interaction between the SA and LAs. The LA (Algorithm 2) provide constraints abstracting the space of solutions of a module and the SA (Algorithm 1) translates and propagates the constraints to the appropriate LAs. The design allows integration of local schedulers without modification, making only minimal assumptions on their behaviour. The approach provides near-optimal results when compared to a monolithic exact scheduler (that is not applicable to modular setups). This is the best achievable result given that the modular approach lacks a comprehensive view of the scheduling process. It offers a good trade-off between scheduling speed and schedule quality and it scales to large production lines. The framework has the potential to integrate different schedulers, thereby combining their strengths and harnessing the benefits of modularity. We have shown the successful integration of two state-of-the-art schedulers (BHCS (van Pinxten et al., 2017) and MNEH (Jeong & Kim, 2014)) into our MAS environment. It provides a solution when monolithic scheduling is not feasible (e.g., in production lines integrating equipment from different vendors). Our MAS framework enables factory designers to assemble customized production lines using available modules and their schedulers, provided that each module implements a compatible LA. Once LAs has been implemented, it is straightforward to reconfigure a production line scheduler. Only the configuration and handover times need to be provided to the SA.

Future research should aim to extend the framework to accommodate more types of production lines. Being able to handle assembly production lines would give a lot more freedom on the type of topologies that are possible. This would allow the merging or splitting of products as well as handling parallel modules. Realizing such an approach requires significant changes in the SA to track the flow of jobs in the production facility, as well as modifications to the propagation strategy to make sure that the algorithm terminates and produces a feasible overall schedule. Another interesting extension would be towards covering production lines that do not require a fixed output order of products. Also such an extension would allow many more scenarios to be covered, since there are many production scenarios where the output order is not significant. But also this extension needs adaptation of the system agent. The SA then needs to achieve consensus on both the processing order of jobs on the various modules and the handover times between modules. Again, termination of the approach and soundness of the obtained global schedules will be the main challenges, besides achieving competitive makespans. It would also be interesting to investigate different, richer abstractions for module behaviour, e.g., abstractions that capture all timing constraints between inputs and outputs of a module, instead of only bounds. This would potentially improve schedule quality at the cost of scheduling time. Such extensions would enhance the versatility and applicability of the framework.

## Data Availability

We provide the source code to generate the experimental data, to run our MAS, and to obtain the experimental results in GitHub (https://github.com/TUE-EE-ES/modular-flow-shop-scheduler) and Zenodo (https://zenodo.org/records/15306150) with DOI https://doi.org/10.5281/zenodo.15306150.

## Footnotes

1 We use the notation ${{\,\textrm{dom}\,}}(f)$ to denote the domain of function $f$.

2 We assume that $\mathsf{si}_{i,k}$ and $\mathsf{dd}_{i,k}$ return 0 resp. $\infty$ if they are undefined.
